# Targeting inhibition of the inflammatory response: advances in the treatment of myocardial fibrosis with natural medicine and active ingredients

**DOI:** 10.3389/fcvm.2025.1627255

**Published:** 2025-08-13

**Authors:** Chang Shu, Chunzhen Ren, Qilin Chen, Yanyun Wang, Ruochen Li, Yue Zhang, Shuyu Yang, Ci Wang, Yingdong Li

**Affiliations:** ^1^ School of Traditional Chinese and Western Medicine, Gansu University of Chinese Medicine, Lanzhou, Gansu, China; ^2^ Department of Traditional Chinese Medicine, Tianjin University of Traditional Chinese Medicine, Tianjin, China; ^3^ First Teaching Hospital of Tianjin University of Traditional Chinese Medicine, Tianjin, China

**Keywords:** myocardial fibrosis, inflammatory response, pathological mechanism, natural medicine, active ingredients

## Abstract

Myocardial fibrosis is a critical pathological foundation of cardiovascular diseases and a fundamental process underlying myocardial remodeling. The inflammatory response is a key driver of myocardial fibrosis, promoting the proliferation of myocardial fibroblasts and collagen deposition through the activation of multiple cytokines and signaling pathways. Natural medicines and active ingredients exhibit distinct therapeutic advantages characterized by not only potent pharmacological efficacy and favorable safety profiles, but also unique multi-target mechanisms of action that enable synergistic modulation of complex pathological pathways. These properties enable them to comprehensively modulate the inflammatory response, thereby providing novel therapeutic strategies for myocardial fibrosis. In this review, we explore the etiology of myocardial fibrosis and the mechanisms by which natural medicines and active ingredients inhibit the inflammatory response to treat myocardial fibrosis. We aim to provide a solid reference for future research on natural medicine-based therapies targeting the inflammatory pathways involved in myocardial fibrosis.

## 1 Background

Cardiovascular disease (CVD) is a major global health problem and is currently the leading cause of death worldwide. In the United States, the prevalence of CVD is as high as 48.6% in adults over 20 years of age, and the prevalence increases with age (1). Myocardial fibrosis (MF) is one of the main manifestations of myocardial remodeling, which is often accompanied by atrial dilatation, cardiomyocyte hypertrophy, and cardiomyocyte apoptosis, and other cardiac manifestations, and has important pathophysiological significance in the development of CVD (2, 3). Myocardial fibrosis is characterized by abnormal proliferation of extracellular matrix fibroblasts, excessive collagen deposition and abnormal distribution (4), and its pathological process involves a variety of mechanisms such as inflammatory response, oxidative stress (OS), ferroptosis and mRNA. Among them, inflammatory response, as a key driver in the process of myocardial fibrosis, plays an important influence in the development of myocardial fibrosis.

The inflammatory response of the cardiovascular system is regulated by multiple systems including the immune response system, renin-angiotensin-aldosterone system (RAAS), sympathetic nervous system, nitrosative redox homeostasis, calcium homeostasis, and heme oxygenase system. Systems that integrally regulate and interact with each other (5). The onset of inflammatory response leads to a large accumulation of inflammatory cells, some of which may release a large number of inflammatory factors, which may constitute an inflammatory pathway with other factors and act directly on fibroblasts, activating them into myofibroblasts and activating fibrotic macrophages and lymphocytes, which in turn triggers fibrotic programs in vascular cells and cardiomyocytes. In addition, long-term chronic inflammation may lead to necrosis of cardiomyocytes, triggering reparative fibrosis. However, the pleiotropic nature of inflammatory mediators and the heterogeneity of patients' cardiac remodeling pose a major challenge to the clinical implementation of strategies targeting the inflammatory response. Dissecting the molecular mechanisms by which inflammatory mediators and related signaling pathways regulate myocardial fibrosis is important for improving the survival and quality of life of patients with CVD (6).

Currently, modern medicine mainly adopts the strategy of inhibiting pro-fibrotic signaling in the treatment of myocardial fibrosis, with drugs such as angiotensin converting enzyme inhibitors (ACEIs) and β-blockers. Although these methods have been proved to have better clinical efficacy, they only have a single pathway of action and suffer from the disadvantages of high side effects and high treatment costs. Natural medicine and active ingredients have the characteristics of multi-component, multi-target and multi-level anti-fibrosis, which have their unique advantages for the treatment of different stages and types of myocardial fibrosis (7), and have good potential in the treatment of myocardial fibrosis. Based on the combing of the causes of myocardial fibrosis and the mechanism of inflammatory response and myocardial fibrosis, this review further composes and explores the process and mechanism of natural medicine and active ingredients regulating myocardial fibrosis by reducing the inflammatory response.

## 2 The cause of myocardial fibrosis

The causes of myocardial fibrosis are complex and varied. According to current research, they mainly include inflammation, oxidative stress, radiation factors, ferroptosis, myocardial electrical remodeling, microRNA and other factors, among which inflammation and oxidative stress are the most common. The complexity and variety of causes bring great challenges to clinical research as well as treatment. The following is an analysis and compilation of the causes of myocardial fibrosis.

### 2.1 Inflammatory response

Inflammatory response is a basic pathological process that occurs when biological tissues are subjected to certain stimuli, such as trauma, infection, etc., and is mainly a defense reaction. Inflammatory mediators are activated and induce myocardial fibrosis. This process mainly includes three steps: secretion of inflammatory cells and inflammatory factors after organ and tissue injury; activation of effector cells by inflammatory cells and inflammatory factors, which promotes proliferation and migration of effector cells; and further secretion of extracellular matrix (ECM) by effector cells, which contributes to myocardial remodeling (6). Inflammatory response-induced myocardial fibrosis is closely related to a variety of inflammatory cells, inflammatory factors and signaling pathways.

Inflammatory cells include mast cells, monocytes, lymphocytes, and macrophages. Mast cells can either directly promote the occurrence of myocardial fibrosis or accelerate the process of myocardial fibrosis through degranulation and release of inflammatory factors, fibrosis mediators, histamine, etc. M1 and M2 macrophages exhibit bidirectional regulation in myocardial fibrosis, which can accelerate myocardial fibrosis through pro-inflammatory response on one hand, and slow down the process of myocardial fibrosis through degradation of ECM on the other hand. Inflammatory factors include tumor necrosis factor (TNF), galectin-3 (gal-3), nuclear factor kappa-B (NF-κB) and interleukin (IL), which act on effector cells and promote myocardial fibrosis through multiple pathways. In addition, TGF-β/Smad signaling pathway, JAK2/STAT3 signaling pathway, and P38 MAPK signaling pathway also play important roles in myocardial fibrosis.

### 2.2 Oxidative stress

OS refers to a negative effect produced by free radicals in the body, which is manifested as an imbalance in which oxidative effects are stronger than antioxidative effects in the body (8). There are two sets of antioxidant systems in the body, enzymatic and non-enzymatic antioxidant systems, which lead to OS when they are not sufficient to reduce the oxidative state of the body (9). Organismal reactive oxygen species (ROS) are key substances that initiate, mediate, and regulate the OS process, and they participate in cellular signaling as signaling molecules under physiological conditions, as well as being an important contributor to organismal aging and the generation of disease (10). Studies have shown that OS is one of the major causes of myocardial fibrosis and interacts with other factors.

**Figure 1:**
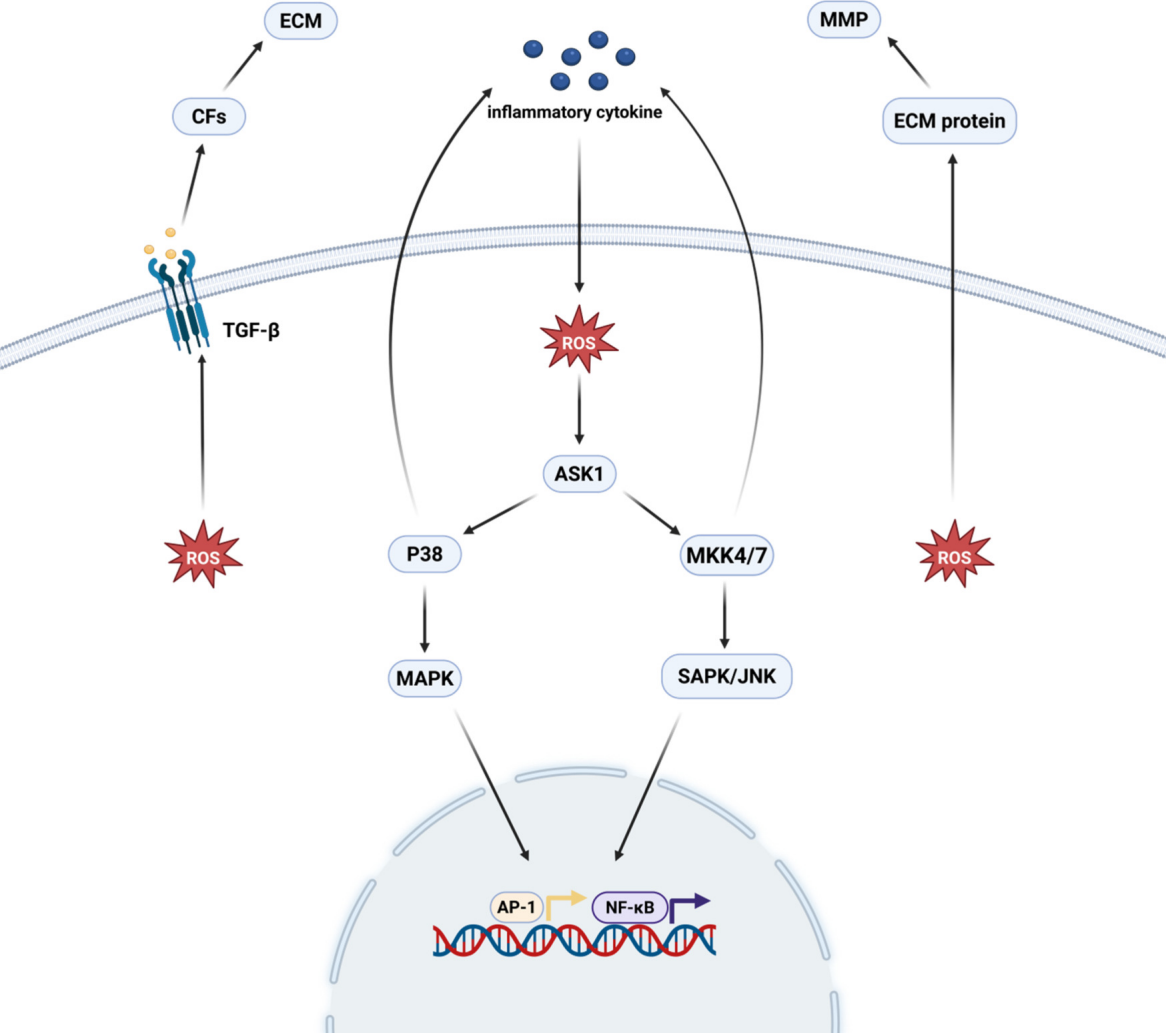
Oxidative stress and myocardial fibrosis. ROS directly regulate MMP in the cardiac interstitium and stimulate the proliferation of cardiac fibroblasts, which promotes the production of TGF-β and thus induces the development of myocardial fibrosis. ROS, after being stimulated by inflammatory factors, promote transcription factors, such as AP-1, ETS/NF-κB, through the activation of the p38 MAPK and SAPK/JNK pathways. activation and enhanced MMP transcription, thereby promoting myocardial fibrosis. Created in BioRender. Yan W (2025) https://BioRender.com/xf62e49.

OS is associated with myocardial fibrosis through direct action as well as participation in cytokine signaling, which promotes the synthesis of pro-fibrotic cytokines, the activation of fibroblasts, and the accumulation of ECM (11). ROS directly regulate the quantity and quality of cardiac mesenchymal MMPs by regulating the expression and metabolism of matrix proteins. And an increase in OS activates MMP and reduces fibrillar collagen synthesis in cardiac fibroblasts (CFs) (12). In addition to its direct effects, ROS can enhance ECM deposition in the cardiac interstitium by activating transforming growth factor-β (TGF-β) (13). In addition, ROS are key mediators in the mechanism of both inflammatory factor and angiotensin II-induced fibroblast action (14). It plays an important role in the activation of mitogen-activated protein kinase (MAPK) and stress-responsive protein kinases by inflammatory factors. Thus, it promotes the activation of transcription factors such as activator protein-1 (AP-1), E-twenty six (Ets) transcription factor and NF-κB and enhances transcription of MMP (15). Meanwhile, angiotensin II activates downstream ROS-sensitive kinase, and this kinase also plays an important role in mediating the process of myocardial fibrosis (16) (Figure 1).

### 2.3 Atrial electrical remodeling

Atrial electrical remodeling refers to changes in the electrophysiologic properties of the atrial muscle, triggered by recurrent episodes of atrial fibrillation (17). It has been pointed out that atrial electrical remodeling can be caused by a variety of factors, such as OS, inflammatory response, and abnormal gene expression, which can also lead to the formation of myocardial fibrosis (18). First, miR-21 expression was significantly elevated in patients with atrial fibrillation, and its expression was equally upregulated as an important factor in the process of myocardial fibrosis and inflammatory response (19). Secondly, inflammatory factors secreted by various inflammatory cells are increased during atrial fibrillation, which can not only cause changes in the expression and function of ion channels and further electrical remodeling of the atria, but also the inflammatory response, which is an important cause of myocardial fibrosis, will likewise have an impact (20). In addition, in the OS state, a large amount of ROS will disrupt ionic pathways and promote atrial electrical remodeling (21), and OS can also aggravate myocardial fibrosis. Overall, atrial electrical remodeling can indirectly affect myocardial fibrosis.

### 2.4 MicroRNA

MicroRNAs (miRNAs) are a class of endogenous, 19–24 nucleotide-long RNAs that were first identified in 1993 in Cryptobacterium hidradii nematodes (22). They function mainly through messenger RNAs, thereby affecting the expression of protein-coding genes (23). Increasingly, miRNAs have been shown to play an important role in the regulation of CVD, including myocardial fibrosis (24). miRNAs can be both pro- and anti-myocardial fibrosis, and whether they promote or inhibit depends on the type of miRNA (25).

MiR-21 and miR-133 mainly play a promotional role in myocardial fibrosis. MiR-21 is closely related to myocardial fibrosis and can affect myocardial fibrosis directly or indirectly from multiple targets. It has been suggested (26) that the three main target genes affecting myocardial fibrosis are SMAD family member 7 (Smad7) and sprouty1/2 (SPRY1/2), and miR-21 can promote myocardial fibrosis by controlling Smad7 and Spry1 (27, 28). In addition, overexpression of miR-21 induces myocardial fibrosis by regulating Jagged 1 and dual-specificity phosphatase 8 (DUSP8), and promotes the transformation of CFs to myofibroblasts and myocardial fibers by targeting Jagged1 (29). MiR-133 is expressed predominantly in cardiomyocytes and myocardial fibroblasts (30), and is involved in cardiomyocyte proliferation, differentiation, hypertrophy growth and other physiological activities, and is closely related to myocardial fibrosis (31).

MiR-29a, miR-30, miR-22, miR-34a, and miR-132 mainly exert inhibitory effects on myocardial fibrosis. MiR-29a, on the one hand, controls the mRNAs encoding the proteins involved in MF, and the overexpression of miR-29a directly leads to the reduction of such proteins (32). On the other hand, miR-29a overexpression inhibits the TGF-β pathway, leading to inhibition of myocardial fibrosis (33). MiR-30 is one of the most abundant miRNAs in the heart, which is released directly from the heart, and is closely related to the collagenvolume fraction (CVF) (34). MiR-30 restricts the key pro fibrotic protein connective tissue growth factor (CTGF) production and directly downregulates the key pro-fibrotic protein CTGF (35). Studies have shown (36) that overexpression of miR-30d improves cardiac function, reduces myocardial fibrosis, and decreases cardiomyocyte apoptosis in rat and mouse models of ischemic heart failure. MiR-22, a miRNA highly enriched in muscle, is also one of the most abundant cardiac miRNAs (37). In several experimental studies (38–41), miR-22 exhibited attenuation of myocardial fibrosis. MiR-34a is a non-negligible regulator in the process of myocardial fibrosis, and it has been experimentally demonstrated that miR-34a can inhibit myocardial fibrosis through multiple pathways (42–45). MiR-132, as a master regulator in the pathological process of heart failure, its role in the inhibition of myocardial fibrosis process, also showed an important role (46–48).

**Figure 2:**
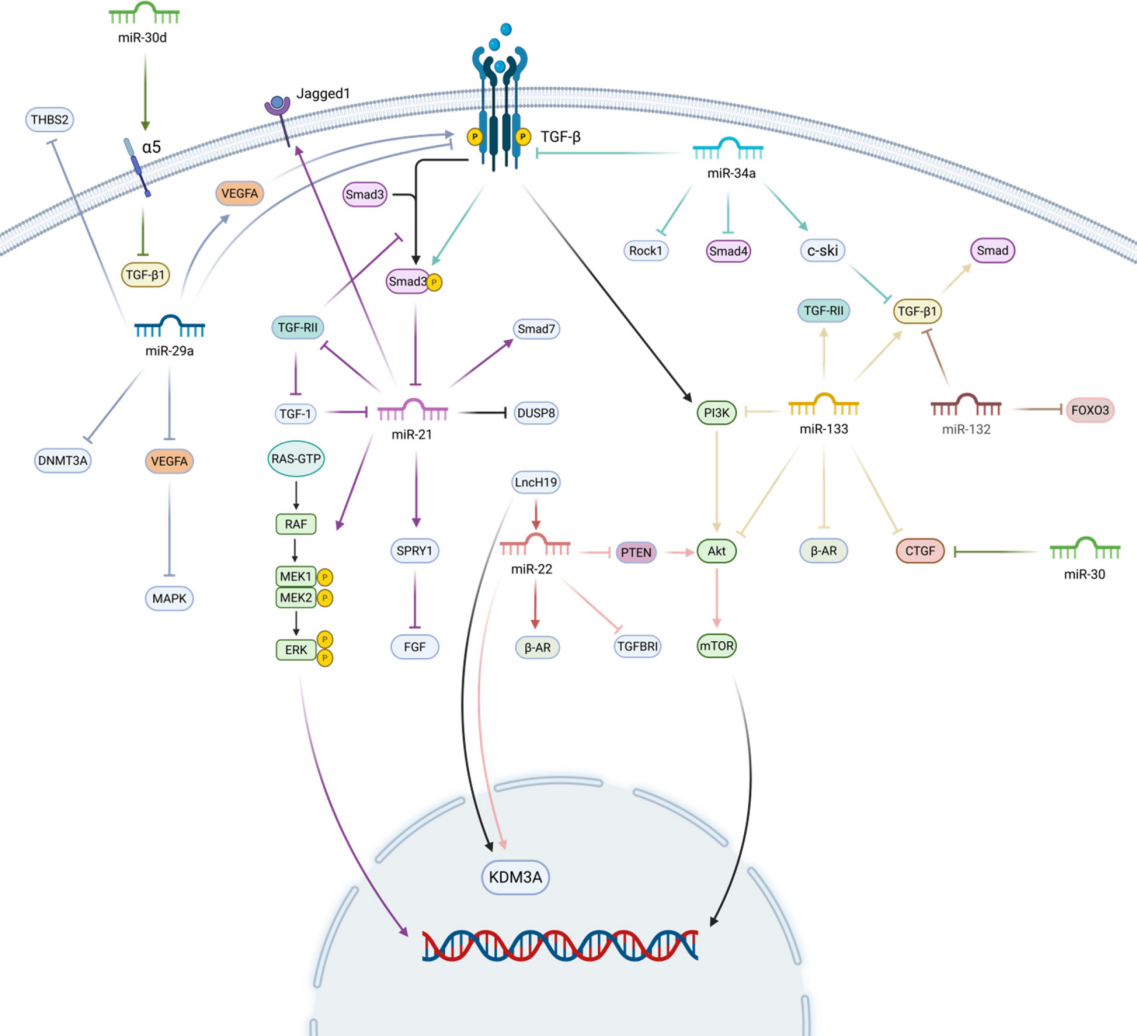
MicroRNAs and myocardial fibrosis. MiR-21 promotes myocardial fibrosis by promoting the expression of Smad7, Spry1, and Jagged 1, and inhibiting the expression of DUSP8. miR-133 mainly activates the TGF-β/Smad pathway and TGF-RII for pro-fibrotic myocardial fibrosis. miR-29a inhibits myocardial fibrosis by inhibiting the expression of TGF-β, vascular endothelial Growth Factor A (VEGFA), and DNA methyltransferase 3A (DNMT3A) to inhibit myocardial fibrosis. miR-30 directly restricts CTGF production and inhibits TGF-β1. miR-22 mainly inhibits the PTEN/Akt/mTOR signalling pathway and directly down-regulates TGFBRI to exert an anti-fibrotic effect. miR-34a and miR-132 inhibit myocardial fibrosis mainly by inhibiting the TGF-β related pathway. Created in BioRender. Yan W (2025) https://BioRender.com/xf62e49.

In summary, due to the numerous types of miRNAs and the diversity of their actions, they can affect myocardial fibrosis through various pathways, which also suggests that we can treat myocardial fibrosis-related diseases from multiple pathways (Figure 2).

### 2.5 Ferroptosis

Iron (Fe) is an essential micronutrient that carries a variety of physiological roles such as transportation and storage of oxygen, mitochondrial respiration and redox reactions (49). The human body contains about 2–5 g of total iron, most of which is bound intracellularly to heme or other non-heme proteins and enzymes in hemoglobin and myoglobin (50, 51). Extracellular iron accounts for only about 0.1% of the total body iron content, and most of which is bound to iron transferrin (TRF) in serum. Disturbances of iron homeostasis include iron overload and iron deficiency. Ferroptosis, an iron-dependent cell death driven by iron accumulation and lipid peroxidation, is characterized by glutathione depletion and inhibition of glutathione peroxidase 4 (GPX4), and has been implicated in the pathogenesis of several CVD (52). Various studies have demonstrated that ferroptosis mediated by iron metabolism imbalance, abnormal lipid peroxidation, reduced GPX4 activity, and inhibition of the cystine/glutamate transport system are closely associated with the development of tissue fibrosis (53, 54). It has now been demonstrated that ferroptosis occurs in ischemic cardiomyocytes and atrial myocytes from patients with atrial fibrillation (55).

Ferroptosis, as a novel type of regulated cell death, is widely involved in the onset and execution of cardiac fibrosis (56). The massive production of lipid ROS during ferroptosis promotes OS, leading to myocardial injury, death, and triggering cardiac fibrosis (57). In addition to promoting the fibrotic process by causing parenchymal cell death, ferroptosis can also promote myocardial fibrosis by inducing inflammation (58). After ferroptosis of cardiomyocyte, alarmins released by necrotic cells triggers a CFS phenotype of pro-inflammatory and matrix-degrading, which may contribute to leukocyte recruitment and activation of the TGF-β cascade response leading to the transformation of fibroblasts into myofibroblasts (59). Meanwhile, the expression of oxidative stress carbonyl protein markers and myocardial fibrosis (type III collagen) was increased in iron-overloaded type 1 diabetic rats (60), suggesting that the process of ferroptosis contributes to the process of myocardial fibrosis by increasing the production of myocardial collagen. Ferroptosis contributes to the development of metabolic diseases, which indirectly leads to increased fibrosis in myocardial tissue through mechanisms such as inflammatory response and OS (61). Furthermore, ferroptosis plays an important role in adriamycin-induced cardiomyopathy, which subsequently triggers myocardial fibrosis (62).

### 2.6 Radiation

Radiation is a specific factor in the induction of myocardial fibrosis. During radiation therapy for chest tumors, it is inevitable that some irradiation damage is caused to the heart, which in turn leads to a variety of serious complications such as myocardial fibrosis, pericardial disease, and damage to the cardiac conduction system. These are referred to as radiation-induced heart disease (RIHD). Radiation-induced myocardial fibrosis (RIMF) accounts for up to 80% of cases (63).

**Figure 3:**
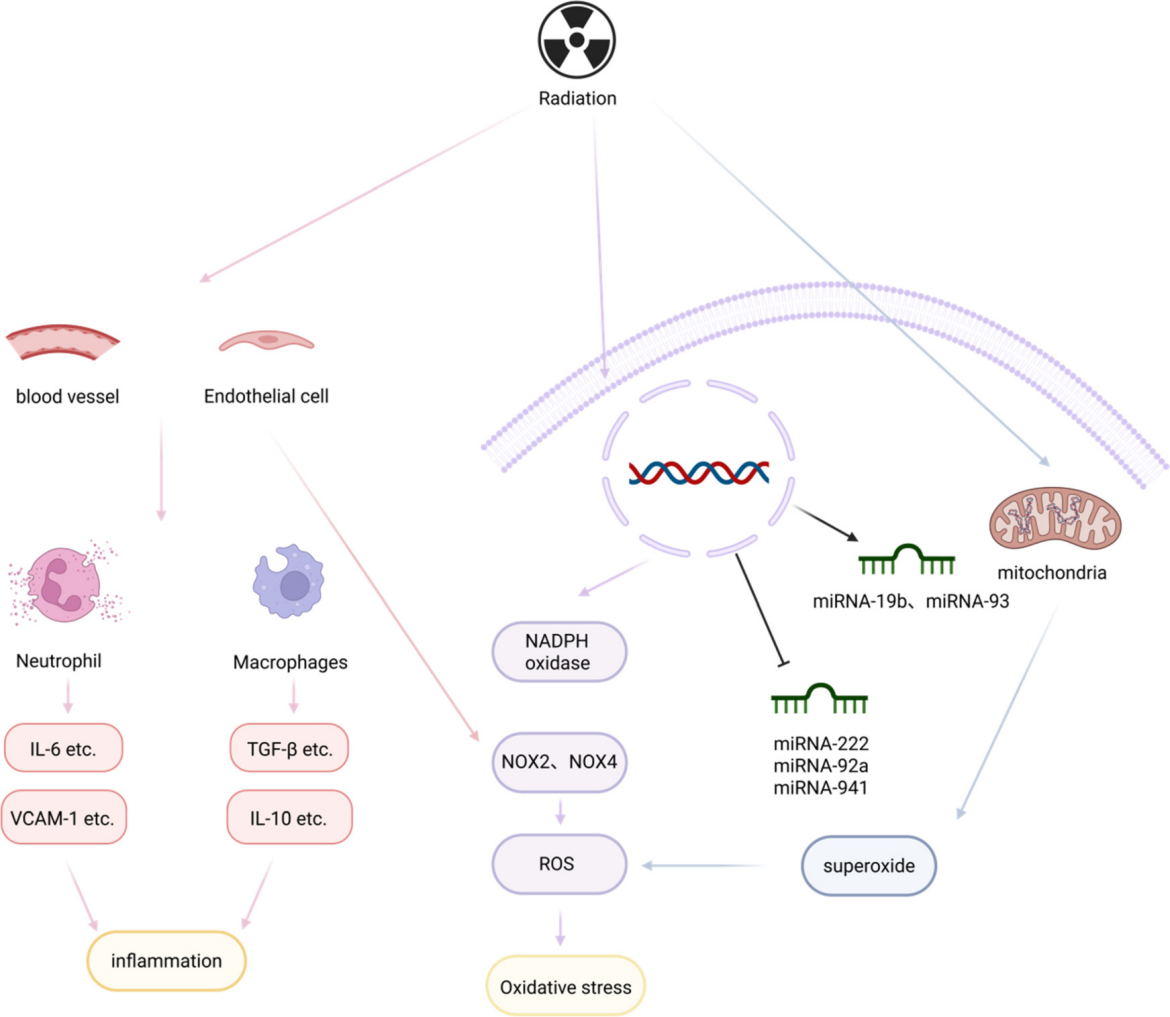
Radiation and myocardial fibrosis. Radiation causes vascular damage and endothelial dysfunction, leading to large aggregations of leukocytes and neutrophils, which in turn secrete inflammatory factors and mediators to mediate the inflammatory response, creating a pro-inflammatory and pro-fibrotic environment. In addition, mitochondrial dysfunction and miRNAs are involved in the slow process of radiation-induced fibrosis, which, by interacting with other factors, leads to the formation of myocardial fibrosis. Created in BioRender. Li R (2025) https://BioRender.com/6beszmg.

Although existing studies have not been able to clearly elucidate the mechanism of radiation-induced myocardial fibrosis, we know that it is a chronic process, and caused by the reciprocal interaction of multiple cytokines and pathways. Radiation first causes vascular injury and endothelial dysfunction, which play a key role in the development of RIMF because it leads to a proinflammatory and profibrotic environment (64–66). Subsequently, radiation can contribute to mitochondrial dysfunction (67) or contribute to the overproduction of ROS *in vivo* by inducing cardiomyocytes and endothelial cells to express high levels of NADPH oxidase 2 (NOX2) and NADPH oxidase 4 (NOX4), which leads to the development of OS (68). In addition, due to the damage of vascular and endothelial, a large number of leukocytes and neutrophils are recruited there, while secreting a large number of factors and inflammatory mediators to mediate the inflammatory response (69, 70). At the same time, miRNAs and the neuroimmune system are involved in the slow process of radiation-induced fibrosis (71, 72), which ultimately leads to the formation of myocardial fibrosis by interacting with other factors. In a sense, radiation, as the etiologic agent, induces the onset of a series of reactions in the body that lead to myocardial fibrosis, and then finally formed myocardial fibrosis (Figure 3).

## 3 Inflammatory response and myocardial fibrosis

### 3.1 Inflammatory cells and myocardial fibrosis

Inflammatory cells play a key role in the inflammatory response by releasing various cytokines and interacting with other cells. The main inflammatory cells involved in myocardial fibrosis include macrophages, mast cells, T lymphocytes and B lymphocytes. In myocardial fibrosis, inflammatory cells promote wound healing and tissue repair on the one hand (73), while on the other hand, they may lead to excessive tissue fibrosis. For example, mast cells tend to be antifibrotic in healthy hearts and promote fibrosis in diseased cardiac tissues (74, 75).

#### 3.1.1 Macrophages

Macrophage (MC) is an immune cell with functions such as immune response, antigen presentation, and phagocytosis, and usually includes classically activated M1 macrophages, as well as alternatively activated M2 macrophages. MC play an important role in various immune processes, inflammatory responses, and fibrosis. Notably, macrophages, as the largest subset of immune cells in the heart (76), are important players in myocardial inflammation and fibrosis (77).

MC promote myocardial fibrosis through multiple pathways. MC directly enhance the survival and activation of myofibroblasts, resulting in increased ECM. In addition, infiltration of mononuclear macrophages is an obligatory process of inflammatory response, and inflammation is a key link in the formation of myocardial fibrosis (78). MC exacerbate the inflammatory response through the secretion of pro-inflammatory factors (e.g., TNF-α, IL-1β, etc.) and the expression of CD86-specific markers (79), while MC produce platelet-derived growth factor (PDGF) and other factors which can activate pro-fibrotic mediators in fibroblasts (80). In addition, after myocardial tissue injury, the associated malfunction of MC will lead to persistent myocardial injury, thus aggravating myocardial fibrosis (81). The malfunctions include abnormal repair, insufficient production of anti-inflammatory MC, and failure of communication between MC and various cells.

Both M1 and M2 macrophages exhibit bidirectional regulation in myocardial fibrosis. In the early stage of cardiac injury, M1 macrophages predominate and produce matrix metalloproteinases (MMPs) that promote ECM degradation, and secrete pro-inflammatory cytokines such as TNF-α and IL-6 that activate CFs (82). The activated CFs increase MMPs while secreting more pro-inflammatory cytokines, further enhancing the inflammatory response (83), which in turn leads to the continuous activation of M1 macrophages and the continuous production of MMPs, which leads to the continuous degradation of the ECM (78). The promotion or inhibition of M1 is mostly related to its mechanism of action, which is different for the M2 type.

In an animal model of angiotensin II-induced myocardial fibrosis, a matrix metalloproteinase-9 (MMP-9) knockout mouse model of myocardial infarction, and a mouse model of aging, M2 macrophages accumulate in large numbers and promote fibroblast activation and collagen synthesis and secretion (84). In animal models such as the IL-13 knockout mouse model of myocardial infarction and the diabetic cardiomyopathy model, M2 macrophages are reduced in injured cardiac tissues, but myocardial fibrosis is aggravated, which in turn suggests an inhibitory effect of M2 macrophages on myocardial fibrosis in these models (85, 86).

In summary, M1 and M2 macrophage make effects on the progression of myocardial fibrosis through multiple pathways.

#### 3.1.2 Mast cells

Mast cells (MC) are innate immune cells that play an important role in innate immune response, acquired immune response, and CVD (87). They are distributed throughout the body, including the heart (88). Activated mast cells can undergo degranulation and release different proinflammatory factors and immunomodulatory mediators into the cellular microenvironment, such as histamine, proteases, and a variety of cytokines, growth factors, and chemokines. Mast cells are closely associated with the inflammatory response while promoting the development of myocardial fibrosis.

Mast cells in the heart promote the development of myocardial fibrosis mainly through degranulation. Mast cell degranulation releases a large number of fibrotic mediators, including histamine, trypsin, chymotrypsin, and various cytokines. Among these factors, TNF-α (89), TGF-β (90), and others are associated with the activation of cardiac fibroblasts. The increase of mast cells in the heart transduces TGF-β-mediated pro-fibrotic signaling, resulting in more pro-fibrotic responses in cardiac fibroblasts (91). In addition, histamine secreted by mast cells stimulates proliferation of fibroblasts and collagen synthesis (92, 93) Tryptase-like enzymes, by activating the protease-activated receptor 2 and inducing the corresponding signaling, result in cardiac fibroblasts with collagen synthesis increase (94). Chymotrypsin, on the other hand, has both synthetic and degradative effects on collagen fibers, promoting angiotensin II (95) production and activation of the TGF1/Smad protein pathway (96), thereby facilitating cardiac fibroblast proliferation and collagen synthesis. At the same time, chymotrypsin activates MMPs and degrades matrix proteins (97). However, it has been experimentally demonstrated that inhibition of chymotrypsin reduces myocardial fibrosis, but chymotrypsin still exerts a pro-fibrotic effect overall (98, 99). In addition to degranulation, mast cells play a unique effect on the development of myocardial fibrosis. In a model of fibrotic cardiomyopathy due to TNF-α overexpression, the development of cardiac fibrosis requires the interaction of mast cells and fibroblasts (91).

#### 3.1.3 CD4+ helper T cells

T lymphocytes and their subpopulations are important components of immunity and are mainly divided into helper T cells (Th cells), regulatory T cells (Tregs cells), and suppressor T cells (Ts cells), and different subpopulations can secrete cytokines to participate in regulation of the inflammatory response (100). The main surface marker of suppressor T cells is CD4, and they are divided into cell subpopulations such as Th1, Th2, Th3, Th9, Th17, and T follicular helper cells, among which Th1, Th2, and Th17 are closely related to myocardial fibrosis.

Th1 cells secrete pro-inflammatory factors such as γ-interferon-γ (IFN-γ), TNF, and interleukin-12 (IL-12). IFN-γ, as a major inflammatory marker, inhibits Th2-mediated activation of fibroblasts and indirectly regulates fibrosis through activation of macrophages (101), and also can inhibit fibrosis through TGF-β-induced Smad3 phosphorylation (102). Meanwhile, Th1 cells can also stimulate the transformation of fibroblasts into collagen fiber-secreting myofibroblasts through direct cell-to-cell interactions (103). Th2 cells secrete factors such as IL-4, IL-5, and IL-13 to promote fibrosis (104). Among them, IL-4 induces the expression of GATA-binding protein 3 in a signal transducer and activator of transcription-6 (STAT-6)-dependent mechanism, which promotes the secretion of IL-4 and IL-5 and inhibits the production of IFN-γ (105). IL-17 secreted by Th17 induces matrix metalloproteinase-1 (MMP-1) production in human cardiac fibroblasts, degrades collagen and mediates tissue remodeling (106, 107).

In summary, CD4+ helper T cells affect myocardial fibrosis according to different cellular subpopulations, and their mechanism of action is mainly through their secreted cytokines.

#### 3.1.4 Bursa dependent lymphocyte

Bursa dependent lymphocyte (B cell) are bone marrow-derived pluripotent stem cells with roles in antibody production, antigen presentation, and immunomodulatory cytokines, which are widely involved in the immune response process (108) and have an important impact on the cardiovascular system (109).

B cells are directly involved in cardiac remodeling through the upregulation of TGF-β1 and IL-6 and produce TNF-α, IL-1β, and IL-6 to maintain a deleterious inflammatory environment. In the study of dilated cardiomyopathy, B cells secreted TNF-α to exert proinflammatory effects and participate in and promote the process of myocardial fibrosis (110). In addition, activated B cells act as antigen presenters, activating CD4+ T cells and promoting their differentiation into the Th1 phenotype (111). Activation of B cells also activates a large number of immune-inflammatory pathways mediated by Toll-like receptors (TLRs), which promotes inflammatory responses and myocardial fibrosis (112). Moreover, activated B cells can recruit inflammatory monocytes Ly6C+ to the myocardium in a Chemokine (C-C motif) ligand 7 (CCL7)-dependent manner, leading to sustained inflammatory progression and myocardial fibrosis (113).

In summary, B lymphocytes can influence myocardial fibrosis by secreting or regulating relevant cytokines, and their activation plays an even more important role.

### 3.2 Inflammatory factors and myocardial fibrosis

#### 3.2.1 TNF-α

Tumor necrosis factor-α (TNF-α), a cytokine with proinflammatory effects, is produced mainly by peripheral macrophages and monocytes, is involved in normal inflammatory and immune responses, and is involved in neutrophil chemotaxis in areas of injury (114). Myocardial fibroblasts secrete TNF-α in response to different types of injury; however, excessive TNF-α secretion leads to myocardial fibrosis.

TNF-α induces myocardial fibrosis through multiple pathways. TNF-α activates transcription factors such as AP1 (115) and WNT1 inducible signaling pathway protein 1 (WISP1) in cardiac fibroblasts, thereby promoting cardiac fibroblast proliferation and collagen secretion, contributing to the development of fibrosis (116). Meanwhile, TNF-α induces OS, which can cause myocardial tissues to overexpress TNF-α, further exacerbating myocardial interstitial fibrosis (117). In addition, TNF-α independently induces upregulation of the angiotensin II type 1 receptor (AT1R), which enhances angiotensin II-mediated pro-fibrotic effects (118). TNF-α also upregulates and activates MMPs, which are responsible for collagen degradation and subsequent matrix deposition, and thus promotes ECM accumulation (119).

Taken together, TNF-α can inhibit myocardial contractility and cause alterations such as fibrosis in cardiomyocytes through a variety of pathways, including activation of endothelial cells, recruitment of inflammatory cells, and increased production of inflammatory cytokines (120).

#### 3.2.2 IL-6

Interleukin-6 (IL-6), IL-6 is a cytokine with multiple activities that is produced by macrophages, vascular smooth muscle cells, and fibroblasts, among others (121, 122). IL-6, as a proinflammatory cytokine, has been implicated in a variety of pathogenetic mechanisms, including inflammation and fibrosis, in CVD (123).

The fibrogenic effects of IL-6 involve direct actions on fibroblasts, as well as indirect effects related to macrophage recruitment, induction of matrix proteins, and up-regulation of growth factors with significant fibroblast-activating properties (like TGF-β) (124). IL-6 is a key component of the proinflammatory effects of calphostin-11 (CDH11) and hypoxia-induced mitogenic factor (HIMF) (125). The fibrogenic effect of IL-6 has been shown to be a major contributor to the inflammation of CVD. IL-6 is a signaling molecule downstream of CDH11 and HIMF, and activate MAPK and calcium-calmodulin dependent protein kinase II (CaMKII) signaling pathway. IL-6 and heat shock protein 90 (Hsp90) synergistically activate the signal transduction and transcriptional activator 3 (STAT-3) signaling pathway (125, 126), leading to excessive collagen synthesis and contributing to the development of myocardial fibrosis (127). In addition, IL-6 can regulate the development of high glucose-induced myocardial fibrosis by enhancing the expression of TGF-β1 and inhibiting the expression of miR-29, which promotes the regulation of myocardial fibroblast proliferation and collagen production (128).

In summary, the inflammatory factor IL-6 promotes the development of myocardial fibrosis by activating inflammatory signaling pathways such as MAPK and STAT3 and activating TGFβ-1 growth factor.

#### 3.2.3 IL-1β

Interleukin-1β (IL-1β), as an isoform of IL-1, is an important pro-inflammatory cytokine, mainly produced by macrophages and monocytes, which plays a key role in the early stage of inflammation and has an important impact on CVD.

Unlike the production mechanisms of most inflammatory cytokines, the production of biologically active IL-1β is dependent on transcriptional, translational, maturation, and secretory mechanisms (129). IL-1β is a member of the nucleotide-binding oligomerization domain (NOD)-like receptor family of pyrin-containing proteins. IL-1β is a downstream inflammatory cytokine secreted by NOD-like receptor family, pyrin domain-containing protein3 (NLRP3) inflammasome, which can utilize the formation of cysteinyl aspartate specific proteinase1 (Caspase1) induced by NLRP3 inflammasome Caspase 1 to mediate the processing and activation of its own precursor to the active form. Activated IL-1β promotes the activation of transcription factors, which drives and enhances the expression of factors such as TGF-β1, IL-4, and IL-13 (130) and promotes myocardial fibrosis. In addition, chronic upregulation of IL-1β activates NLRP3 inflammatory vesicles, whose autocrine signaling to drive differentiation of fibroblasts into myofibroblasts (131). IL-1β also binds to phosphatidylinositol 3-kinase (PI 3) signaling, which through NF-κB upregulates the sustained production and activation of fibroblast growth factor 2 (FGF-2) (132, 133), which promotes endothelial-mesenchymal transition (End-MT), resulting in the gradual loss of endothelial cell morphology and function, and the acquisition of mesenchymal cells, and the loss of endothelial cell morphology and function, which acquires a mesenchymal cell or myofibroblast phenotype (134), regulating the development of myocardial fibrosis after acute myocardial infarction (AMI) (135).

Taken together, IL-1β functions as a factor downstream of NLRP3 inflammatory vesicles and participates in the process of myocardial fibrosis by promoting the production of factors such as TGF-β1 and activating FGF-2 to promote End-MT.

#### 3.2.4 Gal-3

Gal-3, a member of the β-galactan lectin-binding lectin family, is a protein secreted mainly by macrophages, fibroblasts, mast cells, and neutrophils (136). Gal-3 is a biomarker of myocardial fibrosis, is widely expressed in the immune system, and is significantly involved in the process of myocardial fibrosis as a pro-inflammatory and pro-fibrotic molecule.

It has been shown that Gal-3 initially exerts a protective effect in the heart through its anti-apoptotic and anti-necrotic functions, but prolonged expression of this protein leads to the onset of fibrosis (137). Gal-3 enhances macrophage and mast cell infiltration, which promotes the release of inflammatory mediators, such as TGF and IL-1 or IL-2, creating a microenvironment enriched with pro-inflammatory cytokines, thereby promoting fibrosis (138). In addition, Gal-3 is a matricellular glycan-binding protein involved in myocardial fibrosis and remodeling, and activation of Gal-3 leads to its multimerization and formation of Gal-3 lattices on the cell surface, which enhance fibrotic signaling by trapping the TGF-β receptor on the cell surface, and these signaling factors, along with mechanical stresses, promote the transition of quiescent fibroblasts to active, collagen-producing myofibroblasts, thereby inducing the onset of myocardial fibrosis (73, 139, 140). Most importantly, the major binding sites for Gal-3 are located in the extracellular matrix of cardiac fibroblasts and macrophages. Upon myocardial damage, Gal-3 is released at the site of injury and activates resting fibroblasts into matrix-producing fibroblasts by increasing the synthesis of cytoskeletal proteins, such as type I collagen, and inhibiting the activity of MMPs.

In summary, Gal-3 can promote the process of myocardial fibrosis by promoting the development of inflammation, forming Gal-3 lattice to enhance fibrotic signaling, increasing the synthesis of cytoskeletal proteins, and inhibiting the activity of MMPs, and plays an important role in myocardial fibrosis.

#### 3.2.5 IL-16

Interleukin-16 (IL-16) is mainly secreted by T-lymphocytes, epithelial cells, fibroblasts, and monocytes, and has been shown to be a key mediator of several inflammatory, allergic or infectious diseases, as well as playing an important role in promoting myocardial fibrosis (141). Modern studies have shown that IL-16 plays a central role in promoting myocardial fibrosis by prompting the release of TGF-β1 from macrophages infiltrated with cardiomyocytes. In addition, IL-16 enhances the secretion of inflammatory cytokines such as IFN-γ, IL-10, IL-16, TNF-α, and IL-15 by monocytes and mature macrophages, which promotes the development of myocardial inflammation and thus plays an indirect role in promoting the development of myocardial fibrosis (142).

#### 3.2.6 IL-17

Interleukin-17 (IL-17) is a more specific pro-inflammatory cytokine that belongs to a new family of cytokines with no homology to other known interleukins, which are expressed in immune or non-immune cells (143), with IL-17A playing a key role in both myocardial inflammation and myocardial fibrosis. IL-17A specifically acts during the late stage of myocardial remodeling to promote sustained macrophage infiltration and stimulate their production of proinflammatory cytokines, enhancing fibroblast proliferation and pro-fibrotic gene expression (144). In addition, IL-17A increases the expression of MMPs, TIMPs, and collagen, leading to fibroblast migration and myocardial remodeling, resulting in excessive accumulation of interstitial collagen (145).

#### 3.2.7 IL-27

Interleukin-27 (IL-27) is mainly produced by activated antigen-presenting cells such as monocytes, macrophages and dendritic cells (146) and is a member of the Interleukin-12 (IL-12) family, which consists of the α-subunit, IL-27p28, and the β-subunit, Epstein Barr virus induced protein 3 (EBI3) (147). Numerous studies have confirmed that IL-27 not only promotes myocardial fibrosis by acting independently, but also indirectly promotes the process of myocardial fibrosis by activating related signaling pathways. IL-27 regulates the function of fibroblasts and promotes the differentiation of fibroblasts into myofibroblasts, which directly promotes myocardial fibrosis. Meanwhile, IL-27 promotes the activation of Janus kinases (JAKs)/STAT signaling pathway in myocardial fibroblasts, which is involved in the process of myocardial fibrosis (148).

#### 3.2.8 IL-4

Interleukin-4 (IL-4) is an anti-inflammatory cytokine produced by CD4T lymphocytes and mast cells (149, 150). Some experimental studies in mice have shown that IL-4 is an important factor in myocardial cardiac fibrosis in hypertensive hearts (151). In disease states, IL-4 activates mast cells (152), which are pro-fibrotic (153). At the same time, IL-4 produced by mast cells may act in an autocrine manner, leading to further mast cell proliferation and IL-4 production, exacerbating the fibrotic process (154). In addition, IL-4 helps to increase the number of macrophages in fibrotic hearts (155). Moreover, high concentrations of IL-4 can induce macrophage polarization toward an M2 phenotype that inhibits inflammation and promotes scarring (156), a process that has been shown to promote the process of myocardial fibrosis. In addition to this, IL-4 can promote myocardial fibrosis by upregulating collagen genes and stimulating collagen production in mouse CFs, mediated through the signal transducers and activators of transcription 6 (STAT6) signaling pathway (154).

#### 3.2.9 IL-10

Interleukin-10 (IL-10), a multi effector cytokine produced mainly by T helper cells and monocytes/macrophages, has anti-inflammatory effects and promotes the process of tissue healing in injuries caused by infections or inflammation (157). IL-10 is generally believed to inhibit myocardial fibrosis through activation of the STAT3 signaling pathway (158). However, it has been shown that IL-10 produced by myocardial macrophages indirectly activates fibroblasts and stimulates collagen deposition when diastolic dysfunction occurs. Although IL-10 may be beneficial for inflammatory regression and wound healing, it may also have pro-fibrotic deleterious effects in chronic disease settings (159). The *in vivo* effects of IL-10 in the myocardial fibrotic response may depend on the balance between their anti-inflammatory and pro-myocardial fibrotic effects.

#### 3.2.10 IL-33

Interleukin-33 (IL-33), a member of the IL-1 family, is constitutively expressed in the nuclei of endothelial and epithelial cells of normal human tissues (160) and is a biomechanically inducible protein synthesized primarily by CFs (161). The receptor for IL-33 is the growth stimulation expressed gene 2 protein (ST2). ST2 has 2 major isoforms: transmembrane and soluble forms (162). The IL-33/transmembrane ST2l signaling pathway (163), which exerts a protective effect in heart failure, is pro-fibrotic when soluble ST2 binds IL-33 (164, 165). IL-33 is anti-inflammatory and antifibrotic depending on the balance between IL-33 and ST2. In addition to this, IL-33 induces an immune response in Th2 cells, releasing IL-13 and IL-5 (162).

### 3.3 Inflammatory pathways and myocardial fibrosis

Inflammatory response is the main cause of myocardial fibrosis, which is mainly regulated by inflammatory pathways. Inflammatory factors can mediate or be components of inflammatory pathways.

#### 3.3.1 NF-κB

NF-κB is recognized as a classic inflammatory signaling pathway and is widely present in a variety of cells, with a family of five subunits including Rel, RelB, p65 (RelA, NF-κB3), p50 (NF-κB1), and p52 (NF-κB2). NF-κB is involved in the cellular response to inflammatory and stimulatory factors and plays an important role in the pathogenesis of inflammation and fibrosis in the myocardium (166).

The NF-κB signaling pathway promotes the process of myocardial fibrosis through two main pathways. First, NF-κB activation induces the accumulation of inflammatory factors (e.g., TNF-α, IL-1β, IL-6, IL-18, etc.) in myocardial tissues, resulting in increased inflammatory response and consequently myocardial injury, which promotes the proliferation of myocardial fibroblasts and collagen deposition, and ultimately induces the onset of myocardial fibrosis. Second, the homodimer or heterodimer formed by p65 binds to κB protein inhibitors in the cytoplasm, causing IκB degradation and release of NF-κB dimers, which in turn translocates NF-κB into the nucleus, whereas NF-κB, as a central transcriptional effector of inflammatory signaling, will turn on the transcription of target genes (167). In addition, the activation and nuclear translocation of NF-κB can induce the transcription of chemokines [monocyte chemotactic protein-1 (MCP-1)], cytokines (TNF-α, IL-6), and MMPs, and these transcriptionally increased factors greatly contribute to the process of myocardial inflammation and fibrosis through multiple pathways (168).

In summary, NF-κB activation induces the accumulation of other inflammatory factors and nuclear translocation, thereby initiating the transcription of inflammation-related target genes, which play a crucial role in promoting the process of myocardial fibrosis as an important pathway mediating myocardial inflammation.

#### 3.3.2 MAPK

The mitogen-activated protein kinase (MAPK) pathway is a class of phosphorylation-mediated tertiary kinase cascade signaling involved in cell proliferation, differentiation, apoptosis, and fibrosis (169). P38 mitogen-activated protein kinase (p38 MAPK), as the major isoform of the MAPK family, also known as extracellular signal-regulated kinase, regulates inflammatory factors and OS damage *in vivo* (170, 171), and is involved in the pathological process of cellular fibrosis.

The P38 MAPK signaling pathway is activated by the action of angiotensin II (Ang II) on its type 1 receptor, which activates its downstream transcription factors, prompting the massive synthesis and release of TGF-β in cardiomyocytes, leading to the massive proliferation of myocardial fibroblasts, increased expression of collagen, and imbalance of the ratio of type I./III. collagen (172), which ultimately causes myocardial fibrosis. It has been demonstrated that p38 MAPK transduces cytokines and mechanical signals into myofibroblast differentiation through serum response factor (SRF) and calcineurin (CN) (173). In addition, increased levels of p38 in CFs are associated with elevated expression of inflammatory cell-related genes and proteins, and the inflammatory factors produced can exacerbate myocardial fibrosis by activating the p38MAPK pathway in CFs (174).

In summary, the P38MAPK signaling pathway promotes myofibrillar activation to drive the development of myocardial fibrosis by increasing the production of downstream factors such as TGF-β and inflammatory factors.

#### 3.3.3 TGF-β/Smad

Transforming Growth Factor-β (TGF-β) is a multifunctional mediator with three isoforms (TGF-β1, TGF-β2, and TGF-β3) that has been closely associated with a variety of fibrotic processes (175), with TGF-β1 being a key mediator in the development of fibrosis and inflammation. The Smad protein family of Smad2 and Smad3 are two major downstream regulators that promote TGF-β1-mediated tissue fibrosis (176). It has been shown that TGF-β/Smad is a pleiotropic signaling pathway that plays a key role in inflammation and fibrosis, with TGF-β1/Smad considered one of the major pathways inducing the onset of cardiac fibrosis (177).

The promotion of myocardial fibrosis by TGF-β1/Smad is multifaceted. Several studies have shown that TGF-β1 can upregulate the expression of chemokines and proinflammatory factors in both tethered and inflammatory cells, which promotes the development of inflammation, which in turn leads to myocardial fibrosis (178). It has been found that TGF-β mediates inflammation-related myocardial fibrosis can be induced by inducing quiescent fibroblasts to activate and differentiate into extracellular matrix protein-secreting myofibroblasts (179). Among the signaling pathways induced by TGF β, both Smad-dependent and Smad-independent pathways promote cardiac fibrosis (180). Among them, the Smad-independent pathway involves members of the MAPK family. TGF-β stimulates three known MAPK pathways, namely, extracellular signal-regulated kinase, Jun N-terminal kinase, and the p38 pathway (181–183). Transcription factors, which are the primary targets for activation of MAPK, are then stimulated, leading to the initiation of many downstream signaling transductions. These downstream signaling pathways, such as the ERK1/2 signaling pathway, play a pivotal role in inflammation and myocardial fibrosis. In addition, TGF-βs also function in the downregulation of the miR-29 family, and inhibition of the miR-29 family leads to an increase in several key ECM proteins and collagens, which subsequently trigger myocardial fibrosis (172).

In conclusion, the TGF-β/Smad pathway influences the course of myocardial fibrosis either through its pro-inflammatory and contributing pathways to fibroblast differentiation, or through activation of other signaling pathways, modulation of miRNAs, etc.

#### 3.3.4 JAK2/STAT3

The Janus protein tyrosine kinase 2/signal transducer and activator of transcription 3 (JAK2/STAT3) signaling pathway is an important intracellular signal transduction pathway. This pathway is activated in response to protein ligands, including cytokines, growth factors, interferons, and peptide hormones, which in turn regulate a variety of cellular processes, including cell growth, proliferation, differentiation, and apoptosis (184). And it has been closely associated with myocardial fibrosis.

The JAK2/STAT3 pathway is activated by the binding of inflammatory cytokines, such as IL-6, to its specific receptor (185) and stimulates the expression of cytokines, such as IL-6, thereby increasing the inflammatory response and promoting myocardial fibrosis. The activation of the JAK2/STAT3 pathway by resistin up-regulates genes related to fibrosis (186). In addition to this, STAT3 is a key molecular checkpoint for fibroblast activation, which integrates and translates JAK2 kinase activation into a pro-fibrotic response, which in turn induces myofibroblast differentiation and upregulates collagen release (187). In addition, STAT3 is an atypical downstream mediator that transmits the apoptotic effects of TGF-β. Knockdown of STAT3 in fibroblasts prevents TGF-β-induced differentiation of resting fibroblasts to myofibroblasts and significantly reduces the stimulatory effects of TGF-β on collagen release (187). Activation of STAT3 also regulates Angiotensin II (Ang II)-induced cardiac remodeling and is a negative regulator of ventricular hypertrophy and fibrosis (188). Inhibition of STAT3 prevents Ang II-induced fibrosis and cardiac function defects.

Taken together, the JAK2/STAT3 pathway promotes myocardial fibrosis by promoting inflammatory responses, inducing differentiation of myofibroblasts, and transmitting the apoptotic effects of TGF-β.

### 3.4 NLRP3 inflammatory vesicles and myocardial fibrosis

NOD-like receptor protein 3 (NLRP3) is one of the representative inflammatory vesicles of the NOD receptor family and an inflammatory complex protein that has attracted a lot of attention in recent years (189). The NLRP3 inflammasome consists of the receptor NLRP3, the junction protein ASC, and the effector protein pro-caspase-1 (190) and is widely expressed in various cytoplasms. In cardiac fibroblasts, inappropriate activation of NLRP3 inflammatory vesicles can lead to a variety of myocardial dysfunctions including myocardial fibrosis (191).

Under physiological conditions, NLRP3 inflammasome-mediated proinflammatory responses maintain homeostasis and form a protective mechanism for the body (192). Upon myocardial damage, NLRP3 inflammatory vesicles are activated, and after converting pro-caspase-1 to caspase-1, they cleave inflammatory factor precursors, such as pro- IL-1β and pro-IL-18, into mature IL-1β and IL-18 and release them extracellularly. Thereby mediating the development of myocardial inflammation and thus contributing to the onset of myocardial fibrosis (193). Second, NLRP3 inflammatory vesicles activate the TGF-β/Smad and MAPK signaling pathways, which in turn lead to myocardial fibrosis through various pathways, including the promotion of inflammatory factor recruitment (194). In addition, NLRP3 inflammasome activates sterile inflammatory response by recognizing damage-associated molecular pattern (DAMP) in damaged cells (195). And sterile inflammation is closely associated with the levels of pro-inflammatory factors (e.g., TNF-α, IL-6, and IL-1β) that can induce myocardial fibrosis.

**Figure 4:**
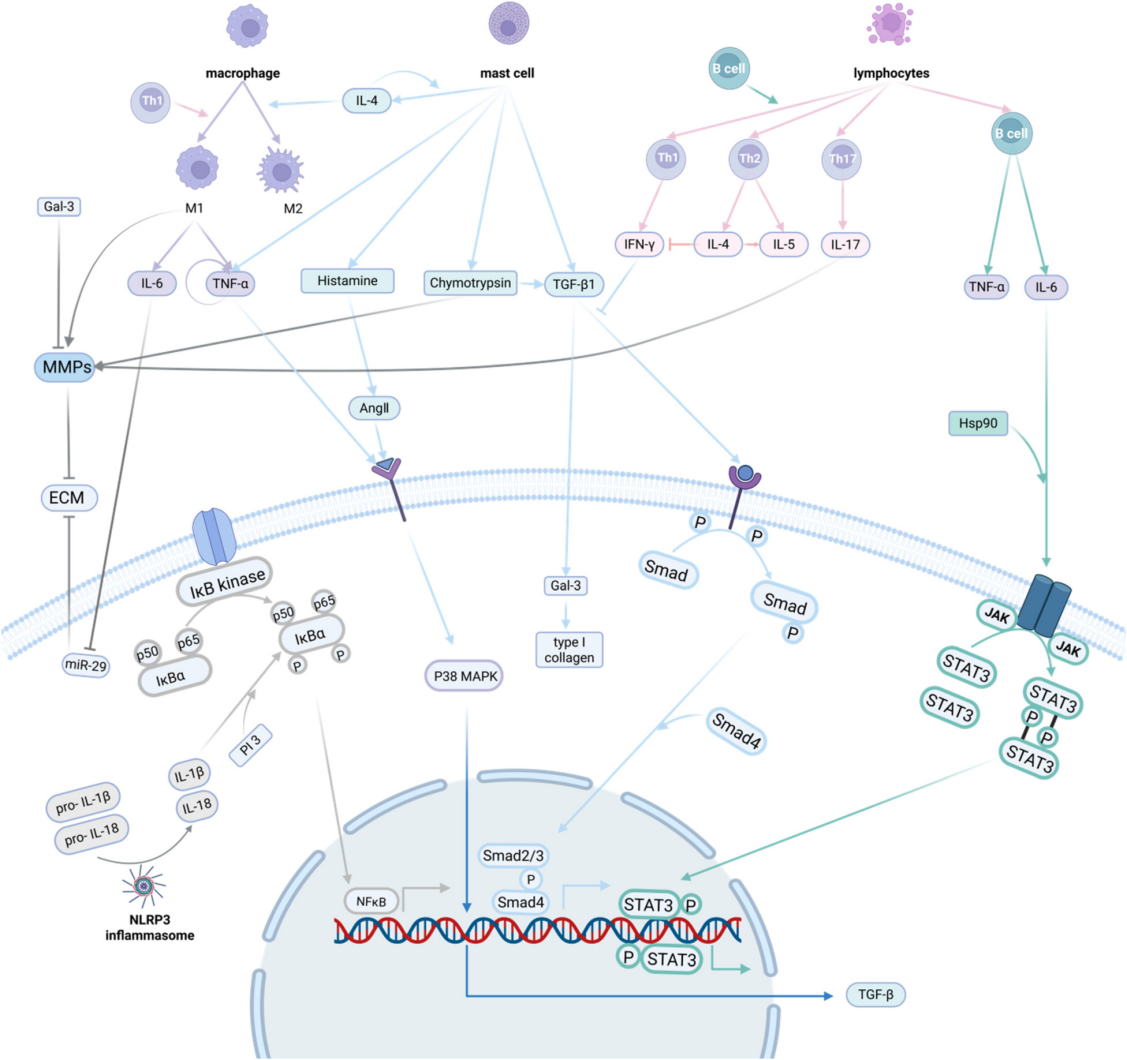
Inflammatory response and myocardial fibrosis. The mechanisms by which the inflammatory response promotes myocardial fibrosis involve multiple aspects of inflammatory cells, inflammatory factors, inflammatory pathways and inflammatory vesicles. Inflammatory cells can secrete inflammatory factors, and inflammatory factors can further activate the inflammatory pathway to act on myocardial fibroblasts, promoting their activation and secretion of extracellular matrix, which in turn promotes the development of myocardial fibrosis. Created in BioRender. Li R (2025) https://BioRender.com/6beszmg.

In summary, NLRP3 inflammatory vesicles affect the development of myocardial fibrosis mainly through three pathways: cleavage and release of mature inflammatory factors, activation of inflammation-related pathways, and activation of sterile inflammatory responses (Figure 4).

## 4 Natural medicine and active ingredients modulate myocardial fibrosis by alleviating the inflammatory response

### 4.1 Traditional Chinese medicine active ingredients

#### 4.1.1 Flavonoids

##### 4.1.1.1 Curcumin

Curcumin is a natural product extracted from turmeric (Curcuma Longa Rhizoma), a plant of the ginger family, and has a variety of antioxidant, anti-inflammatory, anti-tumor, and anti-microbial effects. It reduces inflammation in myocardial fibrosis by inhibiting some signaling pathways, such as MAPKs (196), phosphatidyl-inositol 3-kinase (PI3K) (197), TGF-β (198), and NF-κB (199), and reducing MMP-9 and MMP-2 (200).

Curcumin inhibited Adenosine 5′-monophosphate (AMP)-activated protein kinase (AMPK)/p38 MAPK pathway in rat myocardium by inhibiting TGF-β1 and typical Smad signaling, blocking the synthesis of collagen associated with diabetes (201). Curcumin also reduced the number of advanced glycation end products (AGEs) and the receptor of advanced glycation end products (RAGE) in diabetic rats, thereby inhibiting the activation of MAPKs, PI3K, and NF-κB signaling pathway activation (197). Studies have shown that the combination of curcumin and metformin inhibits JAK/STAT by activating the nuclear factor E2-related factor 2/heme oxygenase-1 (Nrf2/HO-1) pathway and decreasing TGF-β1, thereby attenuating OS and inflammatory responses, which in turn inhibits cardiac fibrosis (202). Also, curcumin inhibits cardiac fibrosis by activating Nrf2, Glutamate-Cysteine Ligase Catalytic Subunit (GCLC), HO-1, and NAD(P)H: quinone oxidoreductase 1 (NQO1) antioxidant effects *in vitro* and *in vivo*, attenuating palmitate-induced ROS increase, inflammation, apoptosis and hypertrophy (201). In addition, curcumin inhibits p38 and c-JunN-terminal kinase (JNK) pathways by increasing the expression of Dickkopf-related protein 3 (DKK-3), which in turn decreases MMP-2 and MMP-9, leading to ECM degradation and vascular degeneration, and attenuates chronic heart failure (CHF) rabbit myocardial fibrosis (202).

##### 4.1.1.2 Licorice glycosides

Glycyrrhizin is a dihydroflavonoid extracted from *Gancao*, which has a variety of pharmacological effects, including antidepressant, anti-inflammatory, antitumor, and cardiovascular protection.

It was found (203), that glycyrrhizin reduced myocardial fiber alignment disorders, inflammatory cell infiltration, and collagen deposition, thereby improving myocardial fibrosis. Liguiritigenin significantly reduced collagen I, collagen III, TGF-β1, MMP-9, α-smooth muscle actin (α-SMA), CCL5, p-NF-κB, TNF-α, and IL-6 expression by inhibiting the expression of CC motif chemokine ligand 5 (CCL5) and NF-κB pathway. Another study (204) also reported that Liquiritin attenuated myocardial fibrosis by reducing the expression of type I collagen, type II collagen, MMP-9, and α-SMA. In addition, another study showed that liquiritin reduced the release of inflammatory cytokines and phosphorylation of NF-κB by inhibiting the IKKα/IκBα signaling pathway.

##### 4.1.1.3 Mangiferin

Mangiferin is a diphenylpyranone flavonoid, which not only exists in mango, but also in a variety of natural medicine, such as *Han Lian Cao*, *Long Dan Cao, Gou Teng*, *Ban Lan Gen*, *Ci Wu Jia Ye*, and *Zhi Mu*. It has a variety of beneficial effects such as anti-tumor, immunomodulation, antioxidant, and anti-inflammatory.

Mangiferin inhibits cardiac collagen deposition while decreasing the levels of cardiac inflammatory cytokines, including IL-1β, IL-6, and TNF-α, as well as the expression of TGF-β1, p-p38, p-MAPK-activated protein kinase 2 (MK2), collagen I (Col-I), collagen III (Col-III) and α-SMA. This suggests that mangiferin inhibits the D-galactose-induced cardiac pro-fibrotic TGF-β1/p38/MK2 signaling pathway, thereby ameliorating cardiac fibrosis (205). Mangiferin inhibits macrophage-associated cytokines, including CD68, monocyte chemotactic protein-1, and TGF-β1, thereby attenuating fructose-induced cardiac interstitial fibrosis (206). Mangiferin also inhibits the p38 MAPK cascade, reducing apoptosis and fibrosis during myocardial remodeling (207). Mangiferin reduces the levels of pro-inflammatory cytokines, pro-apoptotic proteins, and TGF-β, as well as the phosphorylation of p38, thereby alleviating myocardial fibrosis in rats with myocardial ischemia-reperfusion injury (208). In addition, mangiferin reduced the number of inflammatory cells and the area of fibrosis in doxorubicin-induced cardiotoxicity rats (209).

##### 4.1.1.4 Hypericum glycosides

Hypericin, a flavonol glycoside extracted from many traditional natural medicine, has a variety of pharmacological effects including antioxidant, hypoglycemic, anticancer, anti-inflammatory, and cardioprotective.

Hypericin attenuates inflammatory cell infiltration and inhibits the NLRP1 inflammatory pathway by upregulating autophagy (210), thereby inhibiting TGF-β1-induced myofibroblast differentiation and EMC overproduction in neonatal cardiac myofibroblasts by targeting the TGF-β1/Smad signaling pathway (211). Hypericin limits cardiac interstitial fibrosis and inflammatory cell infiltration. The mRNA expression of fibrosis markers, including collagen I, collagen III, and CTGF, the phosphorylation of TGF-β1, Smad2, and Smad3, as well as the expression of IL-1a, IL-6, TNF-α, and MCP-1, were attenuated in the presence of hypericin (212).

##### 4.1.1.5 Biochanin A

Biochanin A is an isoflavonoid found in *Tu Jin Pin*, *Ge Gen*, *San Leng Cao*, and *Deng's Ci Wu Jia*. Many studies have shown that Biochanin A has anti-inflammatory, antioxidant, antimicrobial, and anticancer properties.

Bioflavonoid A significantly reduced the protein levels of collagen III, α-SMA, NLRP3, and p-SMAD, and inhibited the migration and proliferation of fibroblasts (213). Bioflavin A was shown to reduce serum collagen-I, tissue collagen-III, and hydroxyproline levels, resulting in an improvement in isoprenaline-induced myocardial fibrosis. The combination of biotin A and isoprenaline significantly reduced IL-6 expression, whereas brain natriuretic peptide (BNP) and α-SMA were slightly inhibited (214).

#### 4.1.2 Quinones

Tanshinone is a fat-soluble diterpene quinone extracted from the dried roots of *Dan Shen* Bunge. It possesses chemistry activities including antioxidant (215), anti-inflammatory (216), antifibrotic (217), antiviral, antitumor (218), antiplatelet aggregation and neuroprotective (219) ect.

Tanshinone inhibits myocardial fibrosis by inhibiting the phosphorylation of Smad2/3 in rat CFs (220), which reduces the nuclear translocation of Smads and the expression of fibronectin genes (221, 222), as well as decreases the protein levels of fibroblast markers, such as α-SMA, collagen I and III, periosteum proliferating protein and TGF-β, and increases the number of MMP-1 in AngII-treated CFs (223, 224), thereby interfering with the Smad-dependent TGF-β pathway. It was found that myocardial infarction mice with tanshinone administration had fewer necrotic cardiomyocytes at the site of myocardial infarction, regular cellular arrangement, and reduced inflammatory cell infiltration (225). It was also found subsequently that the combination of tanshinone and Puerarin in a 1:1 ratio significantly attenuated acute ischemic cardiomyocyte injury and structure of interstitial edema myocardium, and decreased collagen synthesis and fibroblast release, thereby inhibiting myocardial fibrosis and cardiac remodeling.

### 4.2 Single-ingredient traditional Chinese medicine

#### 4.2.1 Dan shen

*Dan Shen* injection prevents heart failure by attenuating post-infarction remodeling. Yan et al. explored the potential role of the small molecule miR-618 in the anti-myocardial fibrosis of Tanshinone IA, and found that elevated levels of miR-618 could promote the inhibition of cardiac tissue hypertrophy and collagen deposition by Tanshinone IA, and enhance the anti-fibrotic activity of Tanshinone IA (226). The results of the study showed that Tanshinone IA could reduce the incidence of myocardial infarction and enhance myocardial function. Using transcriptome sequencing technology (RNA sequencing, RNA-seg), the study identified 52 gene targets related to myocardial ischemic infarction, of which 21 were inflammation-related genes, and 16 were genes related to the MAPK cascade reaction, and it was further found that salvianolic acid A could reduce the expression of inflammatory factors such as IL-18, IL-6 and TNF-α, increase the quantity of thioredoxin (Trx) and inhibit the activation of JNK pathway, thus inhibiting apoptosis and inflammatory response, and alleviating the effects of myocardial infarction (227). The active ingredients of *Dan Shen* may play an anti-atherosclerosis (AS) role by lowering blood lipids and inhibiting inflammatory response through the TLR4/NF-κB signaling pathway (228). Danhong injection, danshen polyphenate injection, and danshen injection, which are based on the water-soluble components of *Dan Shen*, are also widely used in the emergency treatment of clinical CVD.

#### 4.2.2 Tie Pi Shi Hu

*Tie Pi Shi Hu* attenuates diabetic cardiomyopathy by inhibiting OS, inflammation and fibrosis induced by streptozotocin in mice. By designing a mouse model, it was found that Dendrobium polysaccharides were able to ameliorate functional abnormalities caused by myocardial fibrosis by restoring the activity of aquaporin-5 through the inhibition of lymphocyte infiltration, as well as release of inflammatory factors and apoptosis caused by lymphocyte infiltration (229). In addition, the regulatory effects of Dendrobium polysaccharides on the immune system were investigated in isolated mouse spleens and the RAW264.7 macrophage cell line, and it was found that Dendrobium polysaccharides promoted proliferation of splenocytes, enhanced natural killer cell-mediated cytotoxicity, increased macrophages phagocytosis and production of nitric oxide (NO), and stimulated the secretion of cytokines such as IL-1, IL-2 and TNF-α produced by splenocytes and macrophages (230). The aqueous extract of *Tie Pi Shi Hu* had a cytoprotective effect in an *in vitro* high glucose-induced OS cell model and a lipopolysaccharide-induced cellular inflammation model, which also has dose-dependent manner, and the mechanism of this protective effect may be related to the intracellular OS and inflammatory response through inhibition (231).

#### 4.2.3 Huang jing

The main active ingredient of *Huang Jing* is Polygonatum sibiricum polysaccharide (PSP), which has antiviral, antioxidant, and anti-inflammatory properties, can improve OS levels and inhibit myocardial tissue OS and inflammatory responses, and ameliorate isoproterenol-induced cardiac remodeling in mice (232). It was found (233) that PSP could protect ARPE-19 cells from high glucose-induced OS, inflammation, and apoptosis by inducing the activation of Nrf2/HO-1 signaling pathway. In addition, PSP mitigated the effects of inflammatory cytokines by promoting Nrf2 expression (234). PSP also attenuated diabetic cardiomyopathy in diabetic mice by increasing cyclic guanosine monophosphate-protein kinase G signaling (235). *in vitro* antioxidant activity tests showed that PSP1 had scavenging effects on DPPH, hydroxyl radicals, superoxide anion radicals, and a specific chelating capacity for ferrous iron. This suggests that PSP is useful as a potential antioxidant for the treatment of myocardial fibrosis (236).

#### 4.2.4 Huang Qi

*Huang Qi* and the active ingredients in its formulation, especially astragaloside IV, astragaloside polysaccharides, astragaloside total saponins, astragaloside triterpene saponins, and cycloastragalol, have potential efficacy against MF. It was demonstrated that compared with ischemia-reperfusion-injured rats, Astragaloside IV (ASIV) pretreatment group significantly inhibited malondialdehyde (MDA) levels and induced the Super Oxide Dismutase (SOD) and succinate dehydrogenase (SDH) in myocardial tissues, as well as inhibiting total protein expression of Nrf2 and H0-1 in cardiomyocytes, and decreasing the ratios of p-AKt to AK and p-ERK1/2 to ERK112, suggesting that ASIV exerts its anti-oxidative stress effects through inhibition of the Nrf2/HO-1 pathway (237). It was demonstrated (238) that ASIV inhibited the activation of p38 and JNK signaling pathways while promoting the activation of ERK signaling pathway and prevented high glucose/high fat and hypoxia-induced apoptosis in rat embryonic cardiomyocytes. ASIV decreased adriamycin (ADR)adri-induced Bcl-2-associated X protein/B-cell lymphoma-2 (BaX/BCL-2) ratio and the increase in the number of TUNEL-positive cells, effectively inhibited cardiomyocyte apoptosis. Some experimental results showed that compared with the control group, the left ventricular systolic pressure (LVSP), fractional shortening (FS), and ejection fraction (EF) in the 5 mglkgAs-IV and 10 mgkgAS-V groups were significantly increased, while LVEDP, lactate dehydrogenase (LDH), creatine kinase (CK), heart weight/body weight (HW/BW) ratio and myocardial infarction area were significantly lower (239). This suggests that As-V attenuates myocardial I/R injury in rats through inhibit the PI3K/AKT/glycogen synthase kinase-3 beta (GSK-3B) signaling pathway. There are also findings suggesting that transient receptor potential melastatin 7 (TRPM7) mediated Ca2 + signaling is required for TGF-β induced myocardial fibrosis and could serve as a common pathway in the fibrotic cascade response (240).

### 4.3 Traditional Chinese medicine compound formulas

#### 4.3.1 Compound danshen dripping pill

Compound danshen dripping pill (CDDP) is a kind of proprietary Chinese medicine made by modern medical technology, which is composed of *San Qi*, *Dan Shen* and *Bing Pian*. Tanshinone IA and danshensu contained in *Dan Shen* can dilate blood vessels and reduce myocardial ischemia/reperfusion injury. Ginsenosides and Panax notoginseng saponins in *San Qi* inhibit OS and myocardial fibrosis, and *Bing Pian* has anti-inflammatory and analgesic effects and reduces myocardial oxygen consumption (241, 242). CDDP protects the myocardium through anti-inflammation, anti-oxidative stress, anti-fibrosis, and pro-angiogenesis, and has been widely used in clinical practice for the treatment of coronary artery disease, angina pectoris, and other CVD (243, 244).

Anti-inflammation is the main mechanism by which CDDP exerts cardioprotective effects, and CDDP can inhibits a variety of inflammatory factors and related pathways. Oral CDDP significantly reduced the levels of inflammatory factors such as TNF-α, NF-κB, and IL-6, and ameliorated myocardial injury (245). JNK signaling is a key component of the MAPK pathway, which plays an important role in the inflammatory progression of CVD (246). CDDP inhibits the pro-inflammatory activity of JNK by forming hydrogen-bonded binding to the kinase structural domains of JNK, thereby decreases cardiomyocyte apoptosis (245). In heart-injured mice, both Wnt and lysine-specific demethylase 4A (KDM4A) pathways were significantly activated, inducing inflammation and OS. CDDP exerted anti-inflammatory effects by inhibiting KDM4A activity (247). In addition, CDDP down-regulates forkhead box-O1(FoxO1) and reduces leukocyte adhesion, thereby inhibiting microcirculatory inflammation and improving microvascular function (248).

In summary, CDDP regulates inflammatory response mainly by affecting factors such as TNF-α, NF-κB, IL-6, JNK, KDM4A, and FOXO1, thus inhibiting myocardial fibrosis and improving cardiac function.

#### 4.3.2 Qiliqiangxin

Qiliqiangxin(QL) is extracted from 11 herbs: *Huang Qi*, *Ren Shen*, *Fu Zi*, *Dan Shen*, *Ting Li Zi*, *Ze Xie*, *Gui Zhi*, *Yu Zhu*, *Hong Hua*, *Xiang Jia Pi*, and *Chen Pi*. A multicentre randomised double-blind study confirmed the efficacy of QL in chronic heart failure, and the cardioprotective mechanism of action of QL involves anti-inflammatory, improved energy metabolism and pro-angiogenesis (249–251).

QL regulates microRNAs and a variety of inflammatory factors and plays an important role in attenuating myocardial fibrosis. QL inhibits IL-6 mediated transformation of myocardial fibroblasts, thereby suppressing myocardial fibrosis and cardiac remodeling (252). QL down-regulates the TGF-β1/Smad3 signaling pathway through inhibition of the NLRP3 inflammatory vesicle, thereby suppressing myocardial inflammation and myocardial fibrosis (253). QL also down-regulates the Toll-like receptor 4 (TLR4)/NF-κB signaling pathway and up-regulates the TGF-β3/Smad7 signaling pathway, thereby attenuating cardiac remodeling after myocardial infarction (254). In addition, microRNA regulation is also involved in the anti-fibrotic mechanism of QL. QL inhibits the TGF-β1/Smad3 signaling pathway through up-regulation of miR-133a and miR-345-3p, thereby attenuating myocardial fibrosis and improving cardiac function (255, 256).

In summary, the mechanism of QL treat of myocardial fibrosis is mainly related to the down-regulation of TGF-β1/Smad3, in which inflammatory mediators like NLRP3, miR-133a and miR-345-3p play an important role.

#### 4.3.3 Buyang huanwu decoction

Buyang Huanwu decoction (BYHWD) is a famous formula with a long history, which consists of *Huang Qi*, *Dang Gui*, *Chi Shao*, *Chuan Xiong*, *Tao Ren*, *Hong Hua*, and *Di Long*. BYHWD has been widely used in the prevention and treatment of CVD because of its anti-inflammatory, anti-OS (257), and reparative neurovascular (258) properties.

BYHWD regulates multiple inflammatory factors and inhibits collagen synthesis, playing an important role in inhibiting myocardial fibrosis. BYHWD reduces the release of pro-inflammatory factors through down-regulation of the JAK/STAT pathway, which attenuates left atrial myocardial fibrosis (259, 260). BYHWD down-regulates the expression of MMP9 in rat cardiac fibroblasts, which inhibits inflammatory responses and reduces the proliferation of cardiac fibroblasts (261). BYHWD down-regulated IL-18, NLRP3 inflammatory vesicles, and TLR4/NF-κB signaling pathway by inhibiting the TLR4 signaling pathway, and suppressed the expression level of collagen I/III, thereby attenuating cardiac inflammation and myocardial fibrosis after myocardial infarction (262).

In summary, BYHWD exerts anti-inflammatory effects by down-regulating the expression of inflammatory factors such as IL-6, IL-1β, IL-18, and NLRP3, and inhibiting the JAK/STAT and TLR4/NF-κB signaling pathways, thereby slowing down the process of myocardial fibrosis.

#### 4.3.4 Qi shen Yi Qi pill

Qi Shen Yi Qi pill (QSYQ) is composed of four herbs, *Huang Qi*, *Dan Shen*, *San Qi*, and *Jiang Xiang You*, which have pharmacological effects such as anti-inflammatory, anti-OS, and inhibition of ferroptosis, and are widely used in China for the treatment of CVD such as coronary heart disease and heart failure (263–265).

QSYQ has significant advantages in improving myocardial fibrosis by modulating autophagy and inflammatory pathways. Hyperactivation of autophagy promotes the transformation of fibroblasts to a myofibroblast phenotype, which predisposes to induce myocardial collagen deposition and myocardial fibrosis. Beclin1(BECN1), MAP1LC3(LC3B) (including both LC3-I and LC3-II isoforms) and p62 are the most commonly used autophagy associated markers, and the PI3K/Akt-mammalian target of rapamycin (mTOR) pathway is a key upstream signaling pathway regulating autophagy (266). QSYQ down-regulates Beclin-1 and LC3-II/LC3-I expression, up-regulates p62 expression, and activates the PI3K/AKT-mTOR pathway to dose-dependently inhibit cardiac over-autophagy, thereby slowing down the process of myocardial fibrosis (267). QSYQ inhibited the TGFβ1/Smads signaling pathway and NLRP3 inflammatory vesicle expression, and significantly suppressed monocyte infiltration and macrophage polarization toward M2, thereby inhibiting MMP-2 and MMP-9 expression and ameliorating I/R induced myocardial fibrosis (268, 269). QSYQ down-regulated the TNF-α-NF-κB and IL-6-STAT3 signaling pathways that QSYQ inhibited type I and type II collagen synthesis, thereby improving myocardial remodeling and inhibiting myocardial fibrosis (270). QSYQ inhibited RAAS activation pathway and thereby down-regulated protein expression in the arachidonic acid (AA) metabolic pathway, thereby inhibiting myocardial fibrosis (271).

In summary, QSYQ inhibits NLRP3 inflammatory vesicles and inflammatory signaling pathways such as TGFβ1/Smads, STAT3, and NF-κB, and has multi-target anti-myocardial fibrosis properties.

#### 4.3.5 Gualou xiebai decoction

Gualou Xiebai decoction (GXD) is a long-established Chinese medicinal preparation composed of *Gua Lou* and *Xie Bai*, which contains a variety of compounds that own mechanisms to regulate energy homeostasis and inhibit apoptosis, exerting potential cardioprotective effects (272, 273).

**Figure 5:**
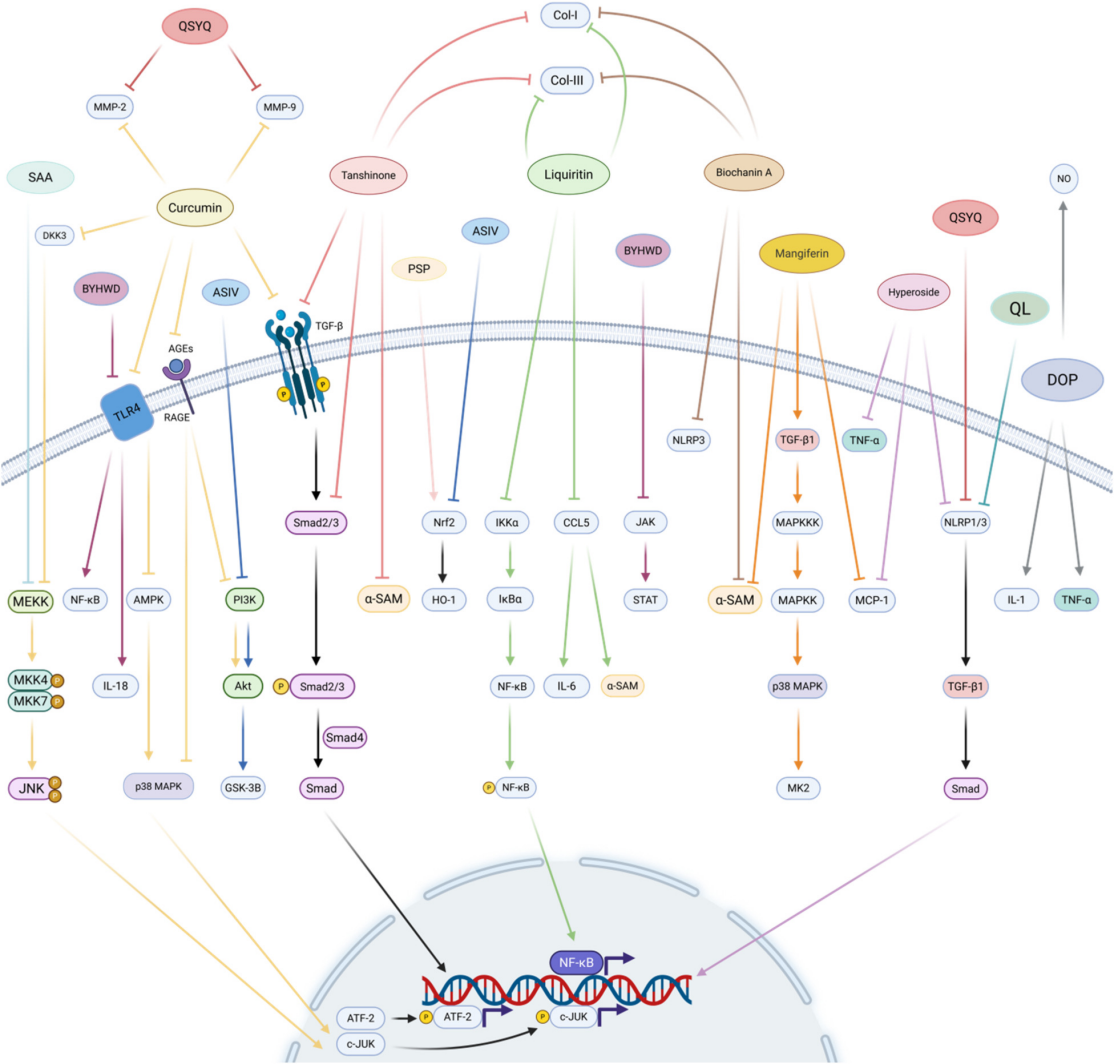
Natural medicine and active ingredients regulate myocardial fibrosis. The active ingredients of Chinese medicines have multi-target and multi-pathway action characteristics, curcumin reduces MMP-2 and MMP-9 by inhibiting JUN, AMPK/p38 MAPK, PI3K, and TGF-β/Smad pathways. tanshinone, glycyrrhizin, and chickpea pigment A are mainly known for inhibiting the TGF-β/Smad signalling pathway, NF-κB phosphorylation and inflammation-related factors, and all three reduced collagen I and collagen III production. Mangiferin and chrysin inhibited myocardial fibrosis by inhibiting the MAPK cascade and TGF-β/Smad signalling pathways, respectively. Single herbs represented by *Dan Shen*, *Tie Pi Shi Hu*, *Huang Jing*, *Huang Qi*, and Chinese herbal medicine combinations represented by Compound Danshen Dripping Pill, Qiliqiangxin, Buyang Huanwu decoction, Qi Shen Yi Qi pill, and Gualou Xiebai decoction, also play an equally important role in the inhibition of myocardial fibrosis. Created in BioRender. Yan W (2025) https://BioRender.com/xf62e49.

GXD mainly exerts anti-inflammatory effects by inhibiting NF-κB and other pathways and related factors, thereby inhibiting the process of myocardial fibrosis. GXD down-regulates the expression of NF-κB target cytokines such as TNF-α, monocyte chemotactic protein-1 (MCP-1), and inhibits the TGFβ1/Smads signaling pathway, thereby inhibiting myocardial type I and type II collagen synthesis and attenuating the cardiac injury cuase by myocardial fibrosis (274, 275) (Figure 5, Table 1).

**Table 1:** Mechanisms by which natural medicines and active ingredients modulate inflammatory responses.

Natural medicine and active ingredients			Prescription composition	Experimental model	Pharmacological effect/mechanisms	References
Traditional Chinese medicine active ingredients	Flavonoids	Curcumin	—	Diabetic rats induced by injection of low-dose STZ in combination with a high-energy diet.	Inhibition of (AMPK)/p38 MAPK pathway in rat myocardium by inhibiting TGF-β1 and typical Smad signaling.	(201)
			—	Diabetic rats induced by injection of low-dose STZ in combination with a high-energy diet.	Reduces the number of AGEs and RAGE, thereby inhibiting the activation of MAPKs, PI3K, and NF-κB signaling pathway.	(197)
			—	Type I diabetic rats induced by injection of STZ.	Combine with metformin can inhibit JAK/STAT by activating the Nrf2/HO-1 pathway and decreasing TGF-β1.	(202)
			—	Diabetic rats induced by injection of low-dose STZ in combination with a high-energy diet.	Activation of Nrf2, GCLC, HO-1, NQO1.	(201)
		Licorice Glycosides	—	Rat MI model established by ligation of the left anterior descending branch of the coronary artery.	Reduced expression of collagen I, collagen III, TGF-β1, MMP-9, α-SMA, CCL5, p-NF-κB, TNF-α and IL-6 by inhibiting the expression of CCL5 and NF-κB pathway.	(203)
			—	Mice with high fructose-induced myocardial fibrosis.	Reduced expression of type I collagen, type II collagen, MMP-9, and α-SMA.	(204)
		Mangiferin	—	Rat model of cardiac fibrosis induced by injected with 150 mg/kg/d Dgalactose for 8 weeks.	Inhibition of the D-galactose-induced cardiac pro-fibrotic TGF-β1/p38/MK2 signaling pathway.	(205)
			—	TAC-induced cardiac fibrosis with impaired cardiac function in mice.	Mangiferin activates Nrf2 and redistributes intracellular glutamate for GSH (glutathione) synthesis, thereby impairing the activation of cardiac fibroblasts as a result of reduced glutamate availability.	(206)
			—	Rat MI model established by ligation of the left anterior descending branch of the coronary artery.	Inhibition of the p38 MAPK cascade.	(207)
			—	IR-induced model of myocardial injury in rats.	Reduced levels of pro-inflammatory cytokines, pro-apoptotic proteins, TGF-β, and the phosphorylation of p38.	(208)
			—	Doxorubicin-induced cardiotoxicity in rats.	Reduction in the number of inflammatory cells and the area of fibrosis.	(209)
		Hypericum Glycosides	—	Rat MI model established by ligation of the left anterior descending branch of the coronary artery.	Inhibition of the NLRP1 inflammatory pathway by upregulating autophagy.	(210)
			—	TGF-β1-induced neonatal rat cardiac fibroblasts.	Inhibit TGF-β1-induced myofibroblast differentiation and EMC overproduction by targeting the TGF-β1/Smad signaling pathway.	(211)
			—	Mice model of mechanical overload-induced cardiac remodeling.	Inhibition of mRNA for fibrosis markers, and expression of IL-1a, IL-6, TNF-α, and MCP-1, and inhibition of phosphorylation of TGF-β1, Smad2 and Smad3.	(212)
		Biochanin A	—	TAC-induced cardiac remodeling in mice.	Reduce the protein levels of collagen III, α-SMA, NLRP3, and p-SMAD, and inhibited the migration and proliferation of fibroblasts.	(213)
			—	ISP-induced cardiac fibrosis in mice.	Combine with isoprenaline significantly reduced IL-6 expression, slightly inhibited BNP and α-SMA.	(214)
	Quinones		—	-	Inhibits the phosphorylation of Smad2/3.	(220)
			—	CFs from newborn rats.	Inhibition of Smad2/3 phosphorylation reduces nuclear translocation of Smads, expression of fibronectin genes and protein levels of fibroblast markers.	(221, 222)
			—	Human adult atrial fibroblasts.	Reduced protein levels of α-SMA, collagen I and III, and fibroblast markers such as TGF-β.	(223)
			—	CFs from newborn rats.	Reduced protein levels of α-SMA, collagen I and III, and fibroblast markers such as TGF-β, and increased the amount of MMP-1 in AngII-treated CFs.	(224)
Single-ingredient traditional Chinese medicine		*Dan Shen*	Tanshinone IIA (TAN)	Induction of CF and rat hearts showing fibrotic features.	The antifibrotic function of TAN is closely related to the function of miRs (which is associated with the upregulation of 101 miRs and the downregulation of 223 miRs): the induction of miR-618 is indispensable for TAN's function against the fibrotic process after heart injury, and the inhibition of miR-618 will weaken the antifibrotic effect of TAN (TAN can inhibit hypertrophy and collagen deposition in heart tissues).	(226)
			Salvianolic acid A (SAL)	TAC-induced MI rats.	SAL treatment reduced inflammatory factors such as IL-1β, IL-6, and TNF-α and decreased tunnel-positive cells and pro-apoptotic Bax after MI. SAL treatment elevated thioredoxin (Trx) and inhibited the activation of c-jun N-terminal kinase (JNK) to attenuate apoptosis and inflammation after MI. SAL protected cardiomyocytes against H2O2-induced H9c2 damage through increasing cell viability, decreasing cell apoptosis, and activating Trx and inhibiting JNK. Taken together, SAL inhibited cell apoptosis and inflammation through Trx/JNK signaling.	(227)
			Danshensu (DSS), salvianolic acid A (Sal-A), salvianolic acid B (Sal-B) and protocatechuic aldehyde (PAL), A mixture of these four ingredients is called SABP.	Male ApoE-/- mice.	SABP may exert an anti-atherosclerotic effect by lowering blood lipids and inhibiting inflammatory response via TLR4/NF-κB signaling pathway.	(228)
		*Tie Pi Shi Hu*	Dendrobium officinale polysaccharide (DOP)	Mice	By designing a mouse model, it was found that Dendrobium polysaccharides were able to ameliorate functional abnormalities caused by myocardial fibrosis by restoring the activity of aquaporin-5 through the inhibition of lymphocyte infiltration, as well as release of inflammatory factors and apoptosis caused by lymphocyte infiltration.	(229)
			Dendrobium polysaccharides.	Isolated mouse spleens and the RAW264.7 macrophage cell line.	The regulatory effects of Dendrobium polysaccharides on the immune system were investigated in isolated mouse spleens and the RAW264.7 macrophage cell line, and it was found that Dendrobium polysaccharides promoted proliferation of splenocytes, enhanced natural killer cell-mediated cytotoxicity, increased macrophages phagocytosis and production of nitric oxide (NO), and stimulated the secretion of cytokines such as IL-1, IL-2 and TNF-α produced by splenocytes and macrophages.	(230)
			The aqueous extract of Dendrobium officinale。	Vitro high glucose-induced OS cell model and lipopolysaccharide-induced cellular inflammation model.	The aqueous extract of Tie Pi Shi Hu had a cytoprotective effect in an *in vitro* high glucose-induced OS cell model and a lipopolysaccharide-induced cellular inflammation model, which also has dosedependent manner, and the mechanism of this protective effect may be related to the intracellular OS and inflammatory response through inhibition.	(231)
		*Huang Jing*	Polygonatum sibiricum polysaccharides (PSP)	Isoprenaline-induced cardiac remodeling in mice.	The main active ingredient of Huang Jing is Polygonatum sibiricum polysaccharide (PSP), which has antiviral, antioxidant, and anti-inflammatory properties, can improve OS levels and inhibit myocardial tissue OS and inflammatory responses, and ameliorate isoproterenol-induced cardiac remodeling in mice.	(232)
			Polygonatum sibiricum polysaccharides (PSP)	ARPE-19 cells	PSP protects ARPE-19 cells from HG-induced oxidative stress, inflammation, and cell apoptosis through regulation of Nrf2/HO-1 signaling pathway.	(233)
			Polygonatum sibiricum polysaccharides (PSP)	3T3-L1 adipocytes	PSP mitigated the effects of inflammatory cytokines by promoting Nrf2 expression.	(234)
			Polygonatum sibiricum polysaccharides (PSP)	Mice fed a high-fat diet for 3 months, followed by intraperitoneal injection of STZ, induced mild hyperglycemia and developed DCM.	PSP can ameliorate DCM conditions in diabetic mice by decreasing ER and oxidative stress, and enhancing cyclic guanosine monophosphate protein kinase G signaling.	(235)
		*Huang Qi*	Astragaloside IV	*in vitro* and *in vivo* I/R induced rat.	Astragaloside IV (ASIV) pretreatment group significantly inhibited malondialdehyde (MDA) levels and induced the Super Oxide Dismutase (SOD) and succinate dehydrogenase (SDH) in myocardial tissues, as well as inhibiting total protein expression of Nrf2 and H0-1 in cardiomyocytes, and decreasing the ratios of p-AKt to AK and p-ERK1/2 to ERK112, suggesting that ASIV exerts its anti-oxidative stress effects through inhibition of the Nrf2/HO-1 pathway.	(237)
			Astragaloside IV (AS-IV)	STZ-induced diabetes in mice after induction of MI.	Astragaloside IV treatment significantly inhibited HG/HF and hypoxia-induced apoptosis of H9c2. AS-IV inhibited activation of JNK and p38 signaling pathway while promoting the activation of EKR signaling pathway. AS-IV treatment rescued cardiac function, suppressed cardiac fibrosis and inflammation, and differently regulated the activation of MAPK signaling pathways.	(238)
			Astragaloside IV	Myocardial I/R in rats.	As-IV can alleviate the myocardial I/R injury in rats through regulating PI3K/AKT/GSK-3β signaling pathways.	(239)
			—	Human atrial fibroblasts.	Transient receptor potential melastatin 7 (TRPM7) mediated Ca2+ signaling is required for TGF-β induced myocardial fibrosis and could serve as a common pathway in the fibrotic cascade response.	(240)
Traditional Chinese medicine compound formulas		Compound Danshen Dripping Pill	*San Qi*, *Dan Shen* and *Bing Pian*	21 RCTs involving 2,356 patients.	Water-solubledanshensu:dilate blood vessels, increass coronary flow, improve microcirculation, reduce platelet aggregation, inhibit fibroblast proliferation and secretion of the matrix, anti-inflammatory Notoginseng saponins:increase coronary blood flow, reduce myocardial oxygen consumption and arterial pressure Dipterocarpaceae:anti-myocardial infarction, reduce myocardial oxygen consumption, anti inflammatory, analgesic effects	(241)
			*Saviae miltiorrhizae Bunge (Lamiaceae), Panax notoginseng Burkill (Araliaceae) and borneol*	Six studies involving 1,051 patients. A rat model of high-altitude hypoxia.	CDDP: inhibite pro-inflammatory cytokines and NF-κB expression, decrease D-dimer, erythrocyte aggregation and blood hemorheology, promote AQP1 and Nrf2 expression.	(243)
			*Radix Salviae (Danshen), Panax notoginseng (Sanqi), and other herbs*	Zebrafish embryos.	CDDP:through VEGF/VEGFR and PI3K/AKT pathway-medi ated angiogenesis.	(244)
			*Salvia miltiorrhiza Bunge (Danshen in Chinese) Panax notoginseng (Burkill) F.H. Chen (Sanqi in Chinese) Borneol (Bingpian in Chinese)*	Acute myocardial ischemic rats.	Sal B:inhibit hyperactive JNK Tanshinol:activate PPARγ, inhibiting NF-κB expression, protect cardio Tanshinol:binding to PPARγ, Sal B:bind to JNK, Tan IIA:target on AKT1 NGR1:target on PI3K synergistically regulating MAPK, PI3K/AKT and PPAR pathways.	(245)
				Apolipoprotein E (ApoE) and LDL receptor (LDLR) dual deficient (ApoELDLR) mice.	CDDP:inhibite Wnt pathway, inhibite KDM4A expression and activity.	(247)
			*Salvia miltiorrhiza* *Panax notoginseng* *borneol*	Control, model and CDDP groups (male mice) Model:a rat model of acute myocardial ischaemia.	CDDP:downregulate the expression of FOXO1 and reduce the leukocyte adhesion molecule CD11b.	(248)
		Qiliqiangxin	*Huang Qi*, *Ren Shen*, *Fu Zi*, *Dan Shen*, *Ting Li Zi*, *Ze Xie*, *Gui Zhi*, *Yu Zhu*, *Hong Hua*, *Xiang Jia Pi*, and *Chen Pi*.	Rats with experimental myocardial infarction.	QL:activate of NRG-1/Akt signaling and suppression of p53 pathway.	(250)
			Radix Astragali, Radix Ginseng, Salvia Miltiorrh iza, etc.	*in vitro* cultured CFs from Sprague-Dawley rats.	QL:reduce IL-6 transcription, regulate nuclear activity of NFAT3.	(252)
			ginseng radix et rhizoma, aconiti lateralis radix preparata, salviae miltiorrhizae radix et rhizoma, descurainiae semen lepidii semen, alismatis rhizoma, cinnamomi ramulus, periplocae cortex, carthami flos, polygonati odorati rhizoma, and citri reticulatae pericarpium (ChengCheng et al.	Rats with HF.	QL: alters the composition of gut microbiota and intestinal barrier functions and exerts potent anti-inflammatory effects by inhibiting the NLRP3 inflammasome activation.	(253)
				A rat model of acute myocardial infarction.	QL:inhibite collagen production, cardiac fibroblast activation, and myofibroblast formation;suppresse the expression of proinflammatory cytokine;suppress TGF-β1/Smad3 and NF-κB signaling.	(254)
				Rats with successful coronary artery ligation surgery.	QLC:increase miR-133a, attenuate TGF-β1, Caspase9, Caspase3, and cleave Caspase3.	(255)
				Rats with doxorubicin-induced congestive heart failure.	QL:reducte in myocardial fibrosis, promote TGF-β3/Smad7, and inhibite TGF-β1/Smad3;reverse Bax/Bcl-2 upregulation;inhibite Smad3 by upregulating miR-345-3p.	(256)
		Buyang Huanwu Decoction	*Huang Qi*, *Dang Gui*, *Chi Shao*, *Chuan Xiong*, *Tao Ren*, *Hong Hua*, and *Di Long*	*in vivo* rats models of middle cerebral artery occlusion and reperfusion (MCAO/R).	Glycosides:regulate the Nrf2-mediated antioxidant stress pathway.	(257)
			—	Ischemic stroke model(rats).	Increase expression of phosphorylated AMPK, cyclic AMP-response element binding protein (CREB) and brain-derive neurotrophic factor (BDNF) accompany by inactivation of the NF-κB.reparative neurovascular.	(258)
			—	Atherosclerosis model.	Glycosides:inhibite the activation of JAK/STAT signaling pathway.	(260)
			*Astragalus membranaceus (Huang Qi), Angelica sinensis (Dang Gui), Radix Paeoniae Rubra (Chi Shao), Lumbricus (Di Long), Ligusticum chuanxiong (Chuan Xiong), Carthamus tinctorius (Hong Hua), and Semen Persicae (Tao Ren)*	A network pharmacology approach.	BYHWD:down-regulate the expression of IL-6, IL-1β, and MMP9 in the IL-17 signaling pathway.	(261)
			*—*	An MI model.	Down-regulate the expression levels of collagen I/III, IL-1β, IL-18 and the TLR4 signalling pathway and the NLRP3 inflammasome.	(262)
		Qi Shen Yi Qi pill	*Radix astragali, Salvia miltiorrhiza, Panax notoginseng, and rosewood*	MI/R model.	Upregulate of SOD and CAT and reducte of NOX gene expression and the subsequent inhibition of oxidative stress, protect mitochondrial morphology and function, preserve ATP and calcium homeostasis.	(263)
			*Astragalus membranaceus (Huangqi), Salvia miltiorrhiza (Danshen), Panax notoginseng (Sanqi), and Dalbergia odorifera (Jiangxiang, DO).*	Rat ascending aortic stenosis (AAS) model.	ASIV and R1 mainly contributing to energy metabolism modulation, DLA to protection of oxidative stress, while DO acting as an adjuvant.	(264)
			—	The cecal ligation and puncture (CLP) experimental sepsis animal model.	Mitigate ferroptosis and vascular barrier damage, reducte oxidative, stressinhibite COX2 and RAGE.	(265)
			*Radix Astragali, Radix Salviae Miltiorrhizae, Radix Notoginseng and Lignum Dalbergiae Odoriferae*	The cardiac myosin-induced rats.	Down-regulate Beclin-1 and LC3-II/LC3-I expression, up-regulate p62 expression, regulate the expression of PI3K/Akt-mTOR pathway-related proteins.	(267)
			—	SD rats.	Regulate autophagy-related proteins, activate PI3K/Akt-mTOR signaling pathway, and inhibite activation and assembly of NLRP3 inflammasome.	(268)
			*Astragalus membranaceus (Huangqi, Fabaceae family), Salvia miltiorrhiza (Danshen, mint family, Lamiaceae), Panax notoginseng (Sanqi, Araliaceae), and Dalbergia odorifera (Jiangxiang, Dalbergia family, Leguminosae)*	Rats with I/R-induced infarct.	Release RPS19 dimer, diminish monocyte migration, infiltration and polarization, reduce the yield of both collagen and MMP-2/MMP-9,and inhibite TGFβ1/TGFβRII/Smad pathway.	(269)
			*Radix Astragali mongolici (“huang-qi” in Chinese) and Salvia miltiorrhizabunge (“dan-shen” in Chinese), Flos Lonicerae, Scrophularia, Radix Aconiti Lateralis Preparata, and Radix Glycyrrhizae*	HF Model rats.	Attenuate the oxidative stress, collagens I and III, MMP-2 and MMP-9.depresse the activations of both TNFa-NF-kB and IL-6-STAT3 pathway, attenuate AngII-NADPH oxidase pathway.	(270)
			*the root of Astragalus membranaceus (Fisch.) Bunge, the root of Salvia miltiorrhiza Bunge, the root and rizhome of Panax notoginseng (Burkill) F.H.Chen and the root of Dalbergia odorifera T.C.Chen.*	HF rat model.	Down-regulate expressions of molecules in RAAS pathway, reduce expressions of JAK1/STAT3, NF-κB and Akt signal transducing proteins.	(271)
		Gualou Xiebai Decoction	-*Gua Lou* and *Xie Bai*	An isoprenaline-induced rat model of chronic myocardial ischemia (CMI).	Regulate energy homeostasis and apoptosis.	(272)
			Trichosanthis Fructus and Allii Macrostemonis Bulbus	*in vivo* rat myocardial infarction model *in vivo* H9c2 cell H/R model.	Activate RISK pathway and attenuating apoptosis.	(273)
				Rats with ligated left anterior descending coronary artery.	GXD:downregulate expressions of TGF-β1, TGFβRI, TGFβRII, Smad2/3 whereas improved Smad7 expression.	(274)
				Rats with myocardial infarction.	Ameliorate the activities of AST, LDH and CK-MB;reduce the increase of inflammatory factors (TNF-α, IL-1β);downregulate the inflammatory mediators (NF-κB p65, TNF-α, MCP-1).	(275)

## 5 Discussion

Inflammatory responses can not only activate multiple inflammatory cells, inflammatory factors, and inflammatory pathways, but also have profound effects on myocardial fibrosis through pathways such as NLRP3 inflammatory vesicles. Therefore, controlling the inflammatory response has become an effective strategy to prevention and cure myocardial fibrosis. In this context, natural medicine and active ingredients of Chinese medicine play a role in promoting angiogenesis and improving myocardial metabolism in CVD by virtue of their multi-target and multi-pathway therapeutic advantages, and their therapeutic efficacy is precise and safe, so they have great advantages and development prospects in the treatment of CVD. Studies have shown that a variety of modern drugs have anti-myocardial fibrosis effects. While natural medicine and active ingredients target the complex molecular mechanisms of myocardial fibrosis, they can act on a variety of cytokines and signaling molecule networks to play a role.

This review comprehensively explains the role of inflammation in the development of myocardial fibrosis from the perspective of inflammation, from the perspective of inflammatory cells, inflammatory factors and inflammatory pathways, etc. This review not only focuses on the single effects of the active ingredients of natural medicine, but also covers the combined effects of single-ingredient traditional Chinese medicine and Chinese medicine compound, providing a comprehensive perspective for clinical and basic research.

The limitation of the review is that there are still significant pharmacological and translational medicine bottlenecks in the current research of natural medicines against myocardial fibrosis. Most TCM active ingredients face serious safety challenges, including dose dependence, hepatotoxicity, electrolyte disorders and risk of drug-drug interactions, e.g., glycyrrhizic acid causes pseudoaldosteronism, curcumin causes elevated liver enzymes when used in high doses, and mangiferin may enhance the toxicity of statins. And its oral bioavailability is generally low, blood concentration is difficult to maintain an effective therapeutic window, such as tanshinone oral absorption rate is low and the peak blood concentration and antifibrotic half of the effective amount of a 10-fold difference. Besides, the experimental drug models in the article are inherently different from human disease: the streptozotocin (STZ)-induced T1DM model of diabetic cardiomyopathy fails to mimic the metabolic profile of clinical T2DM, and there are huge differences between supraphysiologic doses (e.g., 500 mg/kg of curcumin) in animal experiments and tolerated doses in humans. As a review, it cites few clinical studies, and many of the agents discussed still lack long-term toxicity assessments. Further investigations are needed into their potential toxicities and drug–drug interactions.

Additionally, the complexity of the study and the diversity of natural medicine components also bring challenges: ① the complexity of the components of Chinese medicine compound preparations, and the ratio of drugs and the quality of medication will affect the efficacy of treatments, the study is more difficult, so at this stage, although the study has been carried out gradually from the active ingredient to the single-flavored drugs, and then to the Chinese medicine compound, it is still difficult to comprehensively explain all of the mechanisms of its action. ② The preventive effect of natural medicine and active ingredients in the treatment of myocardial fibrosis has to be further studied in clinical experiments and research. ③ Technology continues to progress, research continues to innovate, the come out of new technologies may bring great breakthroughs in the physiological study of myocardial fibrosis, the future of the relevant research still need to be integrated with more cutting-edge, to keep up with the latest research progress, to ensure that the study of the real-time.

In the future, we will focus on exploring the mechanism of natural medicine ingredients inducing cells to produce endogenous protective substances, and investigate in depth how reducing inflammatory response protective effect against myocardial fibrosis. In order to provide data support for the prevention and treatment of myocardial fibrosis by natural medicine and active ingredients, and to provide broader ideas for the development and use of drugs for myocardial fibrosis. Promote the research results translated into clinical use rapidly and provide valuable guidance for the clinical treatment of anti-myocardial fibrosis.
